# Supplementary material for: Dual inhibition of intercellular adhesion molecule-1 and nucleolin reduces RSV infection efficiency in human respiratory organoids
Source: Mol Ther Nucleic Acids. 2026 Apr 17;37(2):102932. doi: 10.1016/j.omtn.2026.102932 (PMC13156738; doi:10.1016/j.omtn.2026.102932)
Supplement: Document S1. Figures S1–S10 and Tables S1–S3 [file mmc1.pdf]

OMTN, Volume *37*

Supplemental information

Dual inhibition of intercellular adhesion molecule-1 and nucleolin reduces RSV infection efficiency in human respiratory organoids

Abeer Keshta, Rina Hashimoto, Yuki Kitai, Yoshitaka Nakata, Ayaka Sakamoto, Shimpei Gotoh, Makoto Takeda, and Kazuo Takayama

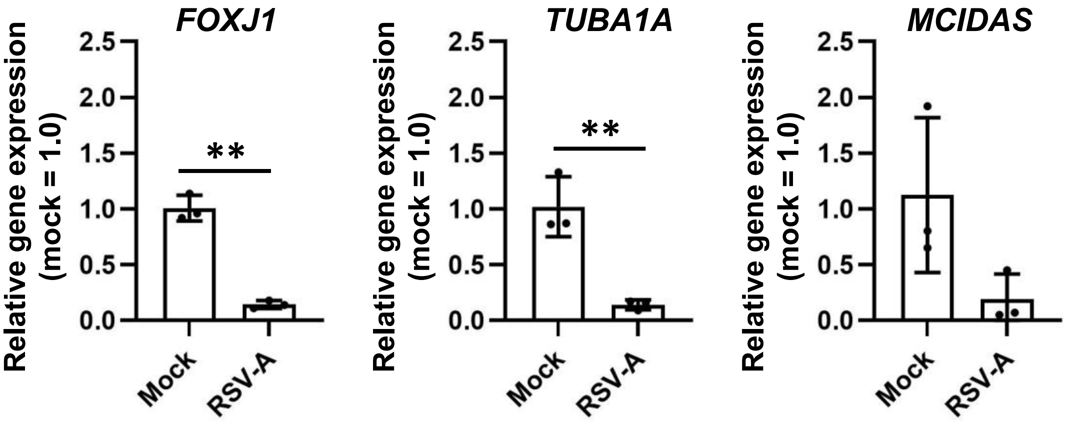

Figure S1. The gene expression levels of airway epithelial cell markers decreased in RSV-A-infected respiratory organoids, related to Figure 1. Human iPSC-derived respiratory organoids were infected with RSV-A at 0.1 TCID/cell (8×10^4 TCID/well) and cultured for 96 h. The expression levels of *FOXJ1*, *TUBA1A*, *and MCIDAS* in respiratory organoids were measured by RT-qPCR (mock = 1.0). Data are shown as mean ± SD (*n*=3, three infection batches); Two-tailed Student's *t*-test (***p* < 0.01).

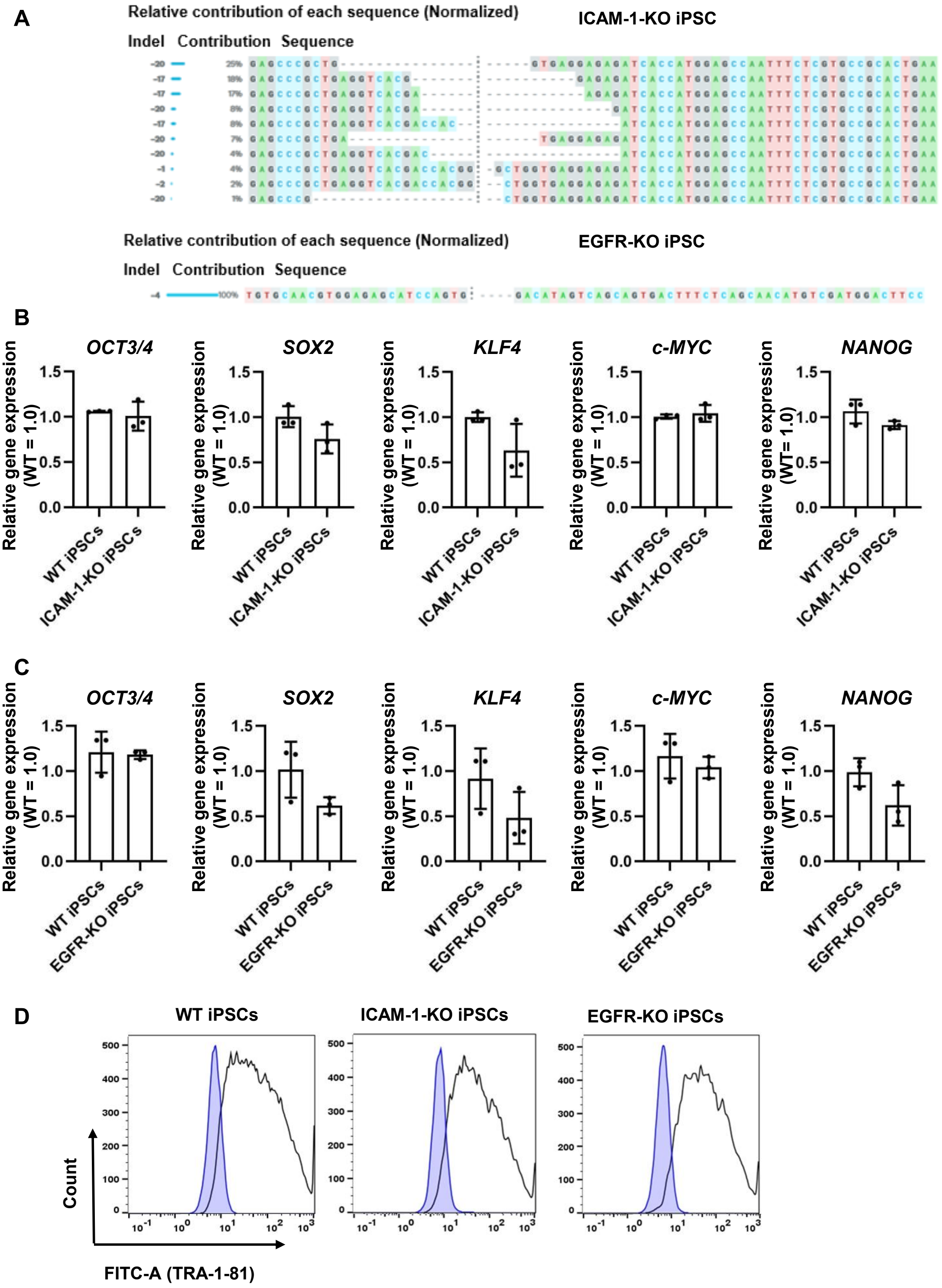

Figure S2. The genome editing efficiency and the expression level of pluripotent markers in ICAM-1- and EGFR-KO iPSCs, related to Figure 2.

(**A**) The indel contribution in each sequence of ICAM-1- or EGFR-KO iPSCs was determined using ICE analysis. (**B**) The gene expression levels of pluripotent

markers, *OCT3/4*, *SOX2*, *KLF4*, *c-MYC*, and *NANOG* in ICAM-1-KO iPSCs were examined by RT-qPCR. Data are shown as mean ± SD (*n*=3, technical replicates). (**C**) The gene expression levels of *OCT3/4*, *SOX2*, *KLF4*, *c-MYC*, and *NANOG* in EGFR-KO iPSCs. Data are shown as mean ± SD (*n*=3, technical replicates). (**D**) The histograms of human iPSCs stained with TRA-1-81 antibody (white) or unstained controls (blue) were obtained by flow cytometry.

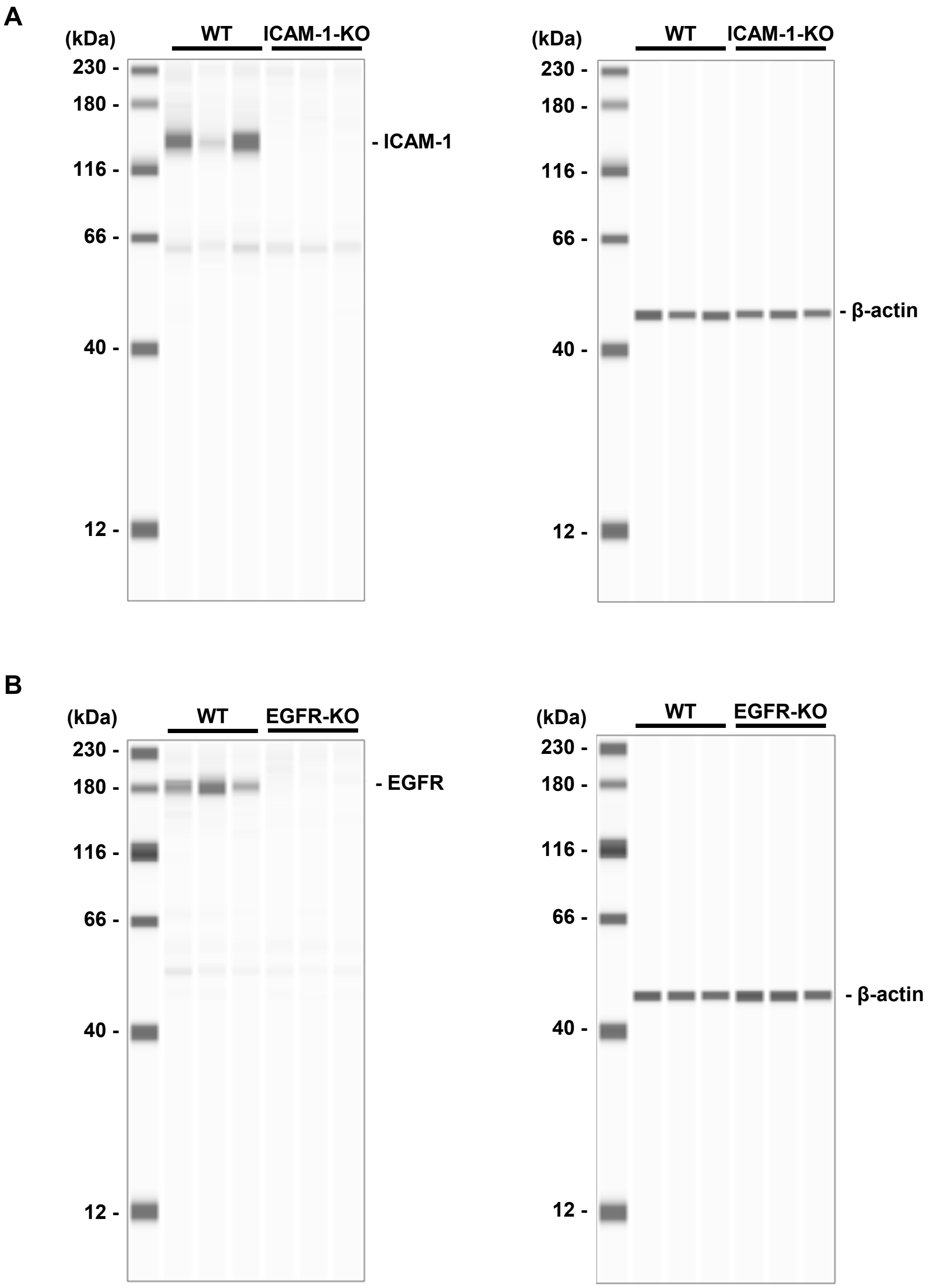

Figure S3. Protein expression analysis in ICAM-1-KO and EGFR-KO respiratory organoids, related to Figure 2.

Full gel images of capillary-based immunoassay of **Figure 2E**. (**A**) ICAM-1 and β-actin expression in ICAM-1-KO respiratory organoids. (**B**) EGFR and β-actin expression in EGFR-KO respiratory organoids.

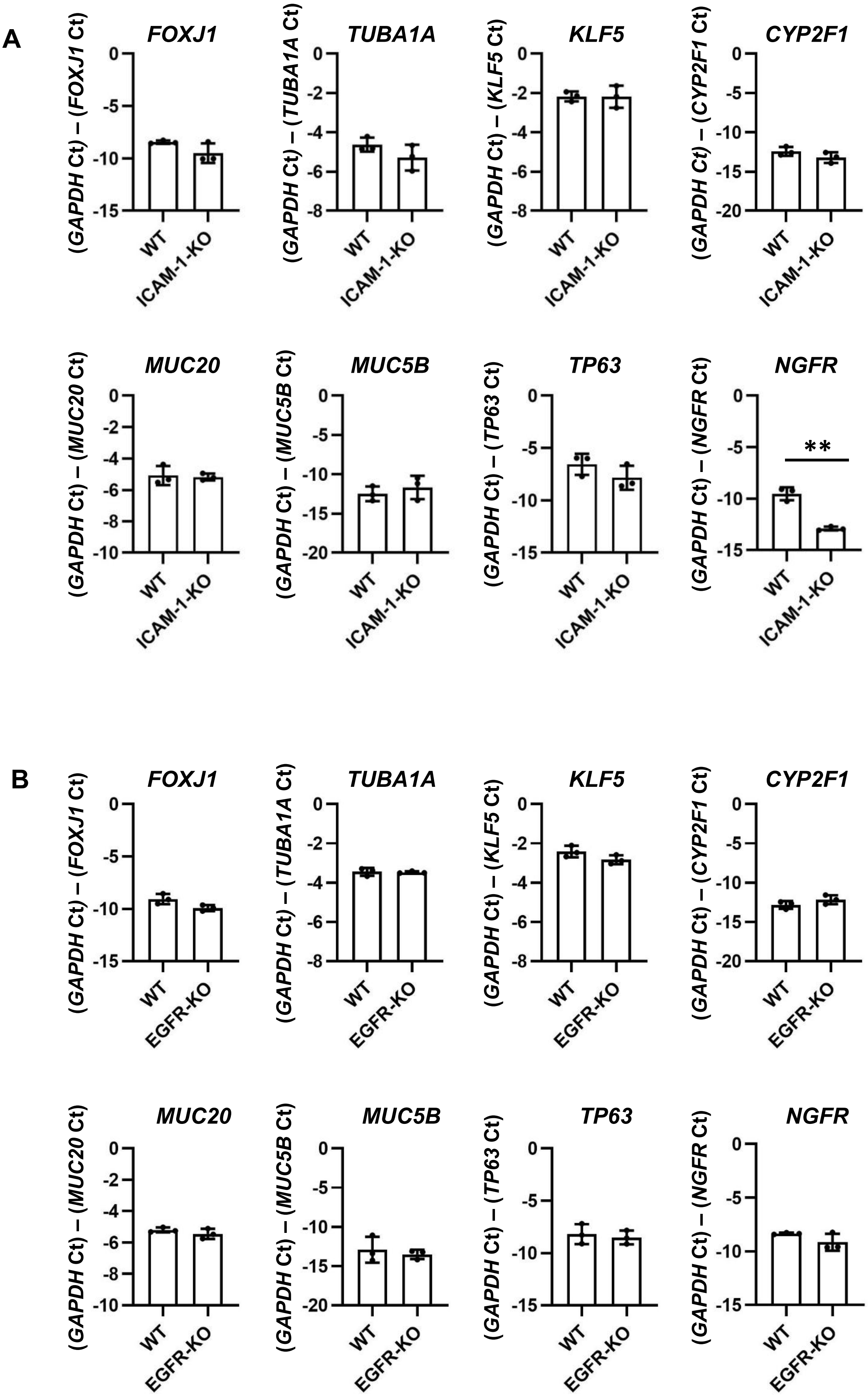
A
FOXJ1
TUBA1A
KLF5
CYP2F1
MUC20
MUC5B
TP63
NGFR
(GAPDH Ct) – (FOXJ1 Ct)
(GAPDH Ct) – (TUBA1A Ct)
(GAPDH Ct) – (KLF5 Ct)
(GAPDH Ct) – (CYP2F1 Ct)
(GAPDH Ct) – (MUC20 Ct)
(GAPDH Ct) – (MUC5B Ct)
(GAPDH Ct) – (TP63 Ct)
(GAPDH Ct) – (NGFR Ct)
WT
ICAM-1-KO
**
B
FOXJ1
TUBA1A
KLF5
CYP2F1
MUC20
MUC5B
TP63
NGFR
WT
EGFR-KO

Figure S4. The gene expression levels of airway epithelial cell markers between WT and KO iPSC-derived respiratory organoids, related to Figure 2.

(**A**) The gene expression levels of ciliated cell markers (*FOXJ1*, *TUBA1A*), goblet cell markers (*MUC20*, *MUC5B*), club cell markers (*KLF5*, *CYP2F1*), and basal cell markers (*TP63*, *NGFR*) in WT and ICAM-1-KO iPSC-derived respiratory organoids were examined by RT-qPCR. Data are shown as mean ± SD (n=3, technical replicates); Two-tailed Student's t-test (**$p < 0.01$). (**B**) The gene expression levels of ciliated cell markers, goblet cell markers, club cell markers, and basal cell markers in WT and EGFR-KO iPSC-derived respiratory organoids were examined by RT-qPCR. Data are shown as mean ± SD (n=3, technical replicates).

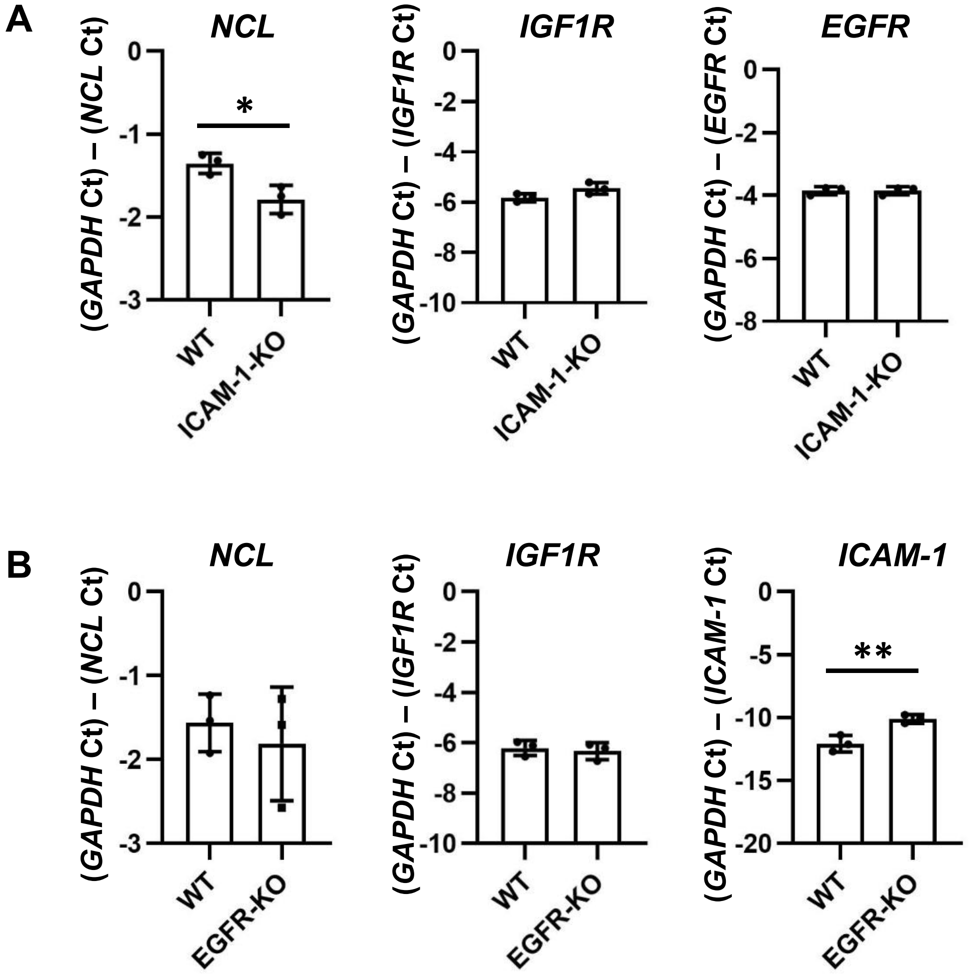

Figure S5. The gene expression levels of RSV F-associated genes between WT and KO iPSC-derived respiratory organoids, related to Figure 2.

(A) The gene expression levels of *NCL*, *IGF1R,* and *EGFR* in WT and ICAM-1-KO iPSC-derived respiratory organoids were examined by PT-qPCR. Data are shown as mean ± SD (*n*=3, technical replicates); Two-tailed Student's *t*-test (**p* < 0.05). (**B**) The gene expression levels of *NCL*, *IGF1R,* and *ICAM-1* in WT and EGFR-KO iPSC-derived respiratory organoids were examined by RT-qPCR. Data are shown as mean ± SD (*n*=3); Two-tailed Student's *t*-test (***p* < 0.01, technical replicates).

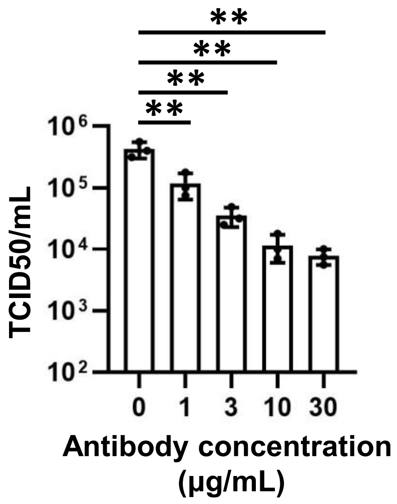

Figure S6. RSV-A infection efficiency decreased in a concentration-dependent manner with increasing levels of neutralizing antibodies, related to Figure 5.

WT iPSC-derived respiratory organoids were treated simultaneously with an anti-NCL antibody and an anti-ICAM-1 antibody. The concentration of each antibody was 0, 1, 3, 10, or 30 μg/mL. After 1 h of incubation, respiratory organoids were infected with RSV-A at 0.1 TCID/cell (8×10^4 TCID/well) and cultured for 96 h. At 96 hpi, the cell culture supernatants of RSV-A-infected WT or antibody-treated respiratory organoids were collected and $TCID_{50}$ assay was performed. Data are shown as mean ± SD (n=3, three infection batches); One-way ANOVA with Tukey's post hoc test ($^{**}p < 0.01$).

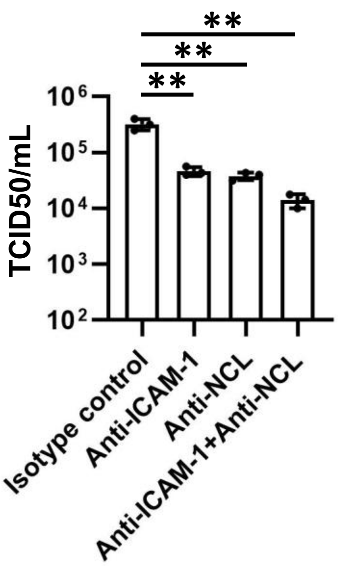

Figure S7. NCL and ICAM-1 also play important roles in RSV-A infection in human ESC-derived respiratory organoids, related to Figure 5.

WT ESC (H9)-derived respiratory organoids were treated with anti-NCL antibodies (10 μg/mL), anti-ICAM-1 antibodies (10 μg/mL), or isotype control (10 μg/mL). After 1 h of incubation, respiratory organoids were infected with RSV-A at 0.1 TCID/cell (8×10^4 TCID/well) and cultured for 96 h. At 96 hpi, the cell culture supernatants of RSV-A-infected WT or antibody-treated respiratory organoids were collected and $TCID_{50}$ assay was performed. Data are shown as mean ± SD (n=3, three infection batches); One-way ANOVA with Tukey's post hoc test (**$p < 0.01$).

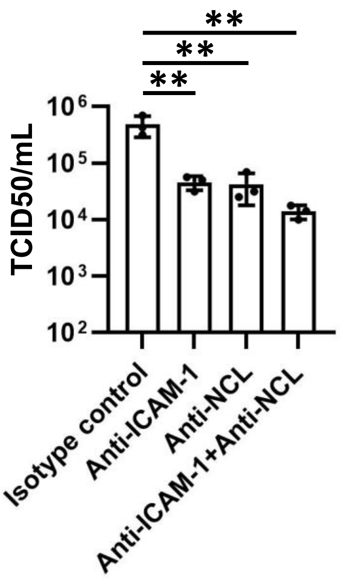

Figure S8. NCL and ICAM-1 also play important roles in RSV-A infection in human ESC-derived respiratory organoids, related to Figure 5.

WT ESC (KhES3)-derived respiratory organoids were treated with anti-NCL antibodies (10 μg/mL), anti-ICAM-1 antibodies (10 μg/mL), or isotype control (10 μg/mL). After 1 h of incubation, respiratory organoids were infected with RSV-A at 0.1 TCID/cell (8×10^4 TCID/well) and cultured for 96 h. At 96 hpi, the cell culture supernatants of RSV-A-infected WT or antibody-treated respiratory organoids were collected and $TCID_{50}$ assay was performed. Data are shown as mean ± SD (n=3, three infection batches); One-way ANOVA with Tukey's post hoc test (**$p <$ 0.01).

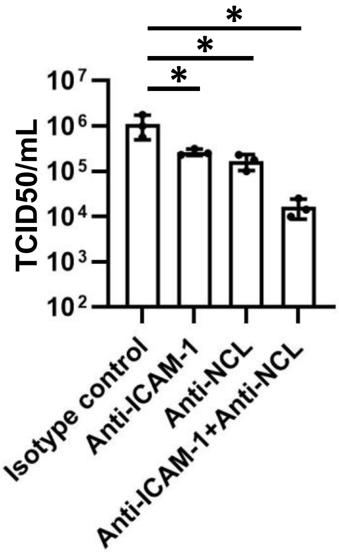

Figure S9. NCL and ICAM-1 play important roles even in high-titer RSV-A infection, related to Figure 5.

WT iPSC-derived respiratory organoids were treated with anti-NCL antibodies (10 μg/mL), anti-ICAM-1 antibodies (10 μg/mL), or isotype control (10 μg/mL). After 1 h of incubation, respiratory organoids were infected with RSV-A at 1.0 TCID/cell (8×10^5 TCID/well) and cultured for 96 h. At 96 hpi, the cell culture supernatants of RSV-A-infected WT or antibody-treated respiratory organoids were collected and $TCID_{50}$ assay was performed. Data are shown as mean ± SD (*n*=3, three infection batches); One-way ANOVA with Tukey's post hoc test (*$p < 0.05$).

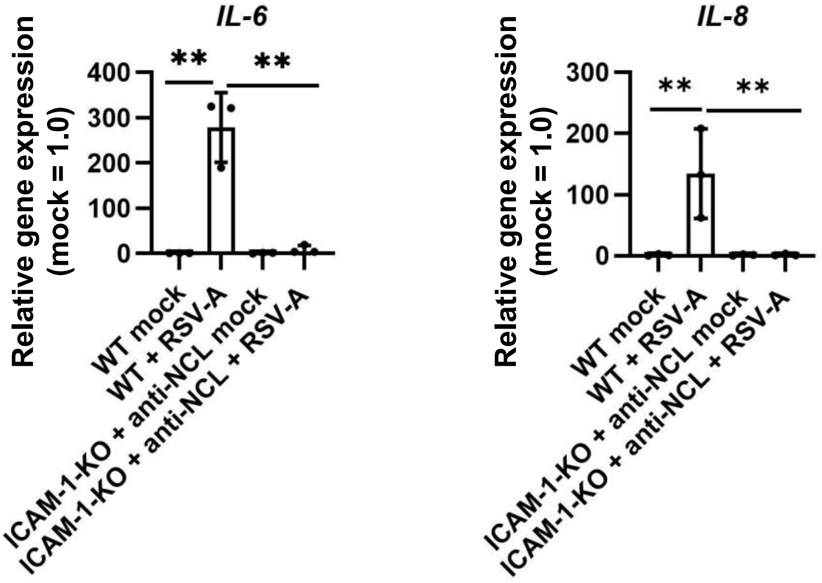

Figure S10. The gene expression levels of inflammatory response-related genes in anti-NCL antibody-treated ICAM-1-KO respiratory organoids, related to Figure 5.

ICAM-1-KO iPSC-derived respiratory organoids were treated with anti-NCL antibodies or an isotype control (10 µg/mL). After 1 h of incubation, respiratory organoids were infected with RSV-A at 0.1 TCID/cell (8×10^4 TCID/well) and cultured for 4 days. At 96 hpi, the gene expression levels of *IL-6* and *IL-8* were measured by RT-qPCR in WT, ICAM-1-KO, and anti-NCL antibody-treated ICAM-1-KO respiratory organoids. Data are shown as mean ± SD (n=3, three infection batches); One-way ANOVA with Tukey's post hoc test (**$p < 0.01$).

Table S1. The sequences of gRNA and primers used for genome editing experiments

ICAM-1	gRNA		TGAGGTCACGACCACGGTGCTGG
	Primers for sequencing	Nested PCR (Forward)	CCAAGATCGAGCCACTGTTT
		Nested PCR (Reverse)	TAGGTGACTGTGGGGTTCAA
		Sequencing	GCGTGTTTGGGGGAGATACT
EGFR	gRNA		TGCTGACTATGTCCCGCCACTGG
	Primers for sequencing	Nested PCR (Forward)	CTGGAGTCTACCTGTCTCTGTC
		Nested PCR (Reverse)	GTAGCCTCTGGAGACTCAGAA
		Sequencing	TGCTCAAAGCCCAATTCAGA

Table S2. Primers used for RT-qPCR

Gene name		Sequence
GAPDH	Forward	GGAGCGAGATCCCTCCAAAAT
	Reverse	GGCTGTTGTCATACTTCTCATGG
ICAM1	Forward	ATGCCCAGACATCTGTGTCC
	Reverse	GGGGTCTCTATGCCCAACAA
EGFR	Forward	CCCACTCATGCTCTACAACCC
	Reverse	TCGCACTTCTTACACTTGCGG
NCL	Forward	GGTGGTCGTTTCCCCAACAAA
	Reverse	GCCAGGTGTGGTAACTGCT
IGF1R	Forward	TCGACATCCGCAACGACTATC
	Reverse	CCAGGGCGTAGTTGTAGAAGAG
MX1	Forward	GTTTCCGAAGTGGACATCGCA
	Reverse	CTGCACAGGTTGTTCTCAGC
ISG15	Forward	CGCAGATCACCCAGAAGATCG
	Reverse	TTCGTCGCATTTGTCCACCA
IL6	Forward	ACTCACCTCTTCAGAACGAATTG
	Reverse	CCATCTTTGGAAGGTTCAGGTTG
IL8	Forward	TTTTGCCAAGGAGTGCTAAAGA
	Reverse	AACCCTCTGCACCCAGTTTTC
FOXJ1	Forward	GCCTCCCTACTCGTATGCCA
	Reverse	GCCGACAGGGTGATCTTGG
TUBA1A	Forward	TCGATATTGAGCGTCCAACCT
	Reverse	CAAAGGCACGTTTGGCATACA
MCIDAS	Forward	ATTCCCACCAAACGGAAGCAG
	Reverse	CCAGGGTAGGCGACATCATAG
KLF5	Forward	CCTGGTCCAGACAAGATGTGA
	Reverse	GAACTGGTCTACGACTGAGGC
CYP2F1	Forward	AGCACAGCCATCTTACTCCTG
	Reverse	GGCAGCTTTCCCTTATCTCTTGA
TP63	Forward	GGACCAGCAGATTCAGAACGG
	Reverse	AGGACACGTCGAAACTGTGC
NGFR	Forward	CCTACGGCTACTACCAGGATG
	Reverse	CACACGGTGTTCTGCTTGT
MUC20	Forward	ATGACAACGGACGACACAGAA
	Reverse	TCAGCGTTTGAGTTTCCAGAG
MUC5B	Forward	GCCTACGAGGACTTCAACGTC
	Reverse	CCTTGATGACAACACGGGTGA
Oct3/4	Forward	CTGGGTTGATCCTCGGACCT
	Reverse	CCATCGGAGTTGCTCTCCA
SOX2	Forward	GCCGAGTGGAAACTTTTGTCG
	Reverse	GGCAGCGTGTACTTATCCTTCT
KLF4	Forward	CCCACATGAAGCGACTTCCC
	Reverse	CAGGTCCAGGAGATCGTTGAA
c-MYC	Forward	GGCTCCTGGCAAAAGGTCA
	Reverse	CTGCGTAGTTGTGCTGATGT
NANOG	Forward	TTTGTGGGCCTGAAGAAAACT
	Reverse	AGGGCTGTCCTGAATAAGCAG
RSV-A N	Forward	CATCCAGCAAATACACCATCCA

	Reverse	TTCTGCACATCATAATTAGGAGTATCAA

Table S3. Antibodies used for immunofluorescence staining, capillary-based immunoassay, or flow cytometry

Antigen	Catalog	Host	Company
Goat anti-Mouse IgG (H+L) Highly Cross-Adsorbed Secondary Antibody, Alexa Fluor™ 594	A11032	Goat	Thermo Fisher Scientific
Respiratory Syncytial Virus	AB43812	Mouse	Abcam
OCT3/4	sc-5279	Mouse	Santa Cruz Biotechnology
β-actin	A5441	Mouse	Sigma-Aldrich
ICAM-1	10831-1-AP	Rabbit	Proteintech Group
EGFR	66455-1-Ig	Mouse	Proteintech Group
TRA-1-81	MAB4381	Mouse	Sigma-Aldrich
