# Supplementary material for: Dual inhibition of intercellular adhesion molecule-1 and nucleolin reduces RSV infection efficiency in human respiratory organoids
Source: Mol Ther Nucleic Acids. 2026 Apr 17;37(2):102932. doi: 10.1016/j.omtn.2026.102932 (PMC13156738; doi:10.1016/j.omtn.2026.102932)
Supplement: Document S2. Article plus supplemental information [file mmc2.pdf]

Molecular Therapy
Nucleic Acids
Original Article

Dual inhibition of intercellular adhesion molecule-1 and nucleolin reduces RSV infection efficiency in human respiratory organoids

Abeer Keshta,[1] Rina Hashimoto,[2] Yuki Kitai,[3] Yoshitaka Nakata,[1,2] Ayaka Sakamoto,[2] Shimpei Gotoh,[1] Makoto Takeda,[3] and Kazuo Takayama[1,2,4]

[1]Center for iPS Cell Research and Application (CiRA), Kyoto University, Kyoto 606-8507, Japan; [2]Department of Synthetic Human Body System, Medical Research Laboratory, Institute of Integrated Research, Institute of Science Tokyo, Tokyo 113-8510, Japan; [3]Department of Microbiology, Graduate School of Medicine and Faculty of Medicine, The University of Tokyo, Tokyo 113-0033, Japan; [4]Department of State-of-the-art and International Medicine, Graduate School of Medicine, Kyoto University, Kyoto 606-8507, Japan

Respiratory syncytial virus (RSV) is one of the major causes of lower respiratory tract infections, particularly in infants and older adults. However, the host factors mediating infection remain poorly defined. It has been suggested that four host surface proteins, namely intercellular adhesion molecule-1 (ICAM-1), epidermal growth factor receptor (EGFR), nucleolin (NCL), and insulin-like growth factor 1 receptor (IGF1R), may interact with the RSV fusion (F) protein. To investigate these roles under physiologically relevant conditions, we employed human induced pluripotent stem cell (iPSC)-derived respiratory organoids as a model for RSV infection. In this model, ICAM-1 and EGFR were genetically depleted using the CRISPR-Cas9 genome editing technique, while NCL and IGF1R were inhibited with neutralizing antibodies. Suppression of ICAM-1 or NCL significantly reduced *RSV* nucleoprotein gene expression, whereas inhibition of EGFR or IGF1R had no observable effect on viral gene expression. Notably, simultaneous suppression of ICAM-1 and NCL resulted in a more substantial reduction in infectious viral titers and RSV F protein expression than inhibition of either protein alone. Our results suggest that both ICAM-1 and NCL may play important roles during RSV infection in human iPSC-derived respiratory organoids.

INTRODUCTION

Respiratory syncytial virus (RSV) is one of the major causes of lower respiratory tract infections, including bronchiolitis and severe pneumonia. It remains a leading driver of hospitalizations in infants[1] and can also cause substantial disease and hospitalization in older adults.[2] Following the decline of coronavirus disease 2019 (COVID-19) restrictions, a marked resurgence of RSV cases has been reported.[3,4] A report in 2023 indicated that the disease burden of RSV is substantial and may exceed that of influenza and COVID-19 in adults aged 60 years and older.[5]

RSV is an enveloped, negative-sense, single-stranded RNA virus. The RSV genome encodes 11 proteins: NS1, NS2, N, P, M, SH, G, F, M2-1, M2-2, and L. Among them, the G and F proteins are major surface glycoproteins that play key roles in viral entry.[6] The G protein mainly mediates viral attachment to respiratory epithelial cells by binding to cellular receptors. The F protein is essential for infection because it promotes fusion between the viral envelope and the host cell membrane, allowing the viral genome to enter the cytoplasm. This fusion requires a conformational change of the F protein.[6]

In recent years, the development and approval of several vaccines (Arexvy, Abrysvo, and mResvia)[7–9] and long-acting monoclonal antibodies (nirsevimab and clesrovimab)[10,11] represent important advances in RSV prevention. Most of these interventions target the viral fusion (F) protein, which plays a central role in mediating viral entry.[6,12] Several host factors, including nucleolin (NCL),[13] intercellular adhesion molecule-1 (ICAM-1),[14] epidermal growth factor receptor (EGFR),[15] and insulin-like growth factor-1 receptor (IGF1R),[16] have been proposed to interact with RSV F protein and facilitate RSV entry. However, it is still unclear which receptor plays the most important role in RSV infection. In addition, it is possible that more than one receptor contributes to infection. Therefore, it is necessary to clarify the relative roles of each receptor and to examine whether multiple receptors are involved in RSV infection at the same time.

Conventional models for RSV infection research, such as cell lines, mice, and cotton rats,[17] have provided valuable insights. However, these systems do not fully reproduce the structural complexity of the human respiratory epithelium or accurately reflect virus-host interactions because of species differences and simplified cellular

Received 3 December 2025; accepted 14 April 2026;
https://doi.org/10.1016/j.omtn.2026.102932.

Correspondence: Kazuo Takayama, Department of Synthetic Human Body System, Medical Research Laboratory, Institute of Integrated Research, Institute of Science Tokyo, Yushima 1-5-45, Bunkyo-ku, Tokyo 113-8510, Japan.
E-mail: takayama.kazuo@tmd.ac.jp

Published by Elsevier Inc. on behalf of The American Society of Gene and Cell Therapy.
This is an open access article under the CC BY license (http://creativecommons.org/licenses/by/4.0/).

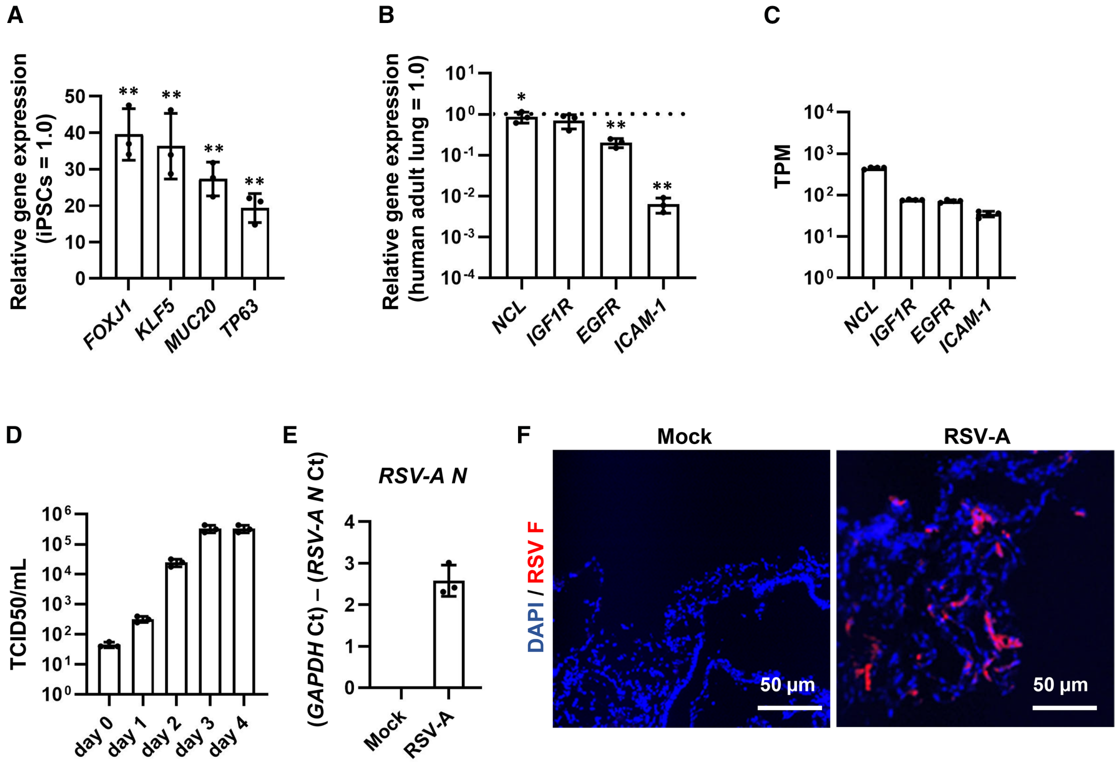

Figure 1. RSV-A efficiently infects human iPSC-derived respiratory organoids

(A) The expression levels of *FOXJ1*, *KLF5*, *MUC20*, and *TP63* in human iPSC-derived respiratory organoids and undifferentiated iPSCs were measured by RT-qPCR (iPSCs = 1.0). Data are shown as mean ± SD ($n = 3$, technical replicates); Two-tailed Student's t test ($^{**}p < 0.01$, compared with iPSCs). (B) The expression levels of *ICAM-1*, *EGFR*, *NCL*, and *IGF1R* in respiratory organoids and human adult lung were measured by RT-qPCR (human adult lung = 1.0). Data are shown as mean ± SD ($n = 3$, technical replicates); Two-tailed Student's t test ($^{*}p < 0.05$, $^{**}p < 0.01$, compared with human adult lung). (C) TPM values of *ICAM-1*, *EGFR*, *NCL*, and *IGF1R* in respiratory organoids. (D–F) Human iPSC-derived respiratory organoids were infected with RSV-A at 0.1 $TCID_{50}$/cell (8×10^4 $TCID_{50}$/well) and cultured for 96 h. (D) The cell culture supernatants of RSV-A-infected respiratory organoids were collected and $TCID_{50}$ assay was performed. Data are shown as mean ± SD ($n = 3$, three infection batches). (E) RSV nucleoprotein (*N*) gene expression was analyzed by RT-qPCR. Data are shown as mean ± SD ($n = 3$, three infection batches). (F) Immunofluorescence staining of RSV fusion (F) protein (red) in infected respiratory organoids at 96 hpi. Nuclei were counterstained with DAPI (blue). Scale bars, 50 μm.

composition. Human respiratory organoids are advanced three-dimensional (3D) models that mimic the architecture and cellular diversity of respiratory tissue. To overcome the limitations of conventional models, we established human induced pluripotent stem cell (iPSC)-derived respiratory organoids containing diverse respiratory epithelial cell types.[18] These organoids have proven to be physiologically relevant models for respiratory virus research, including severe acute respiratory syndrome coronavirus 2 (SARS-CoV-2)[19,20] and RSV.[18]

In this study, we used human iPSC-derived respiratory organoids to evaluate the contributions of NCL, ICAM-1, EGFR, and IGF1R to RSV infection. By generating ICAM-1- and EGFR-knockout (KO) respiratory organoids and using neutralizing antibodies against NCL and IGF1R, we systematically evaluated the relative contribution of each host factor to viral infection. In addition, to examine whether more than one receptor is involved in RSV infection, we also performed experiments using genome-edited respiratory organoids and neutralizing antibodies in combination.

RESULTS

Respiratory organoids were susceptible to RSV

To validate the differentiation of human iPSCs into respiratory epithelial cells, we measured the expression of respiratory epithelial cell markers by quantitative reverse-transcription PCR (RT-qPCR). The expression levels of multiple respiratory epithelial cell markers such as fork-head box protein J1 (*FOXJ1*, ciliated cell marker), krueppel-like factor 5 (*KLF5*, club cell marker), mucin-20 (*MUC20*, goblet cell marker), and tumor protein p63 (*TP63*, basal cell marker) were highly expressed in respiratory organoids (Figure 1A). Next, we examined the expression levels of four host factors—ICAM-1, EGFR, NCL, and IGF1R—that have been reported to be associated with the RSV F protein.[13–16] The expression levels of *NCL* and *IGF1R* in respiratory organoids were comparable to those in the human adult lung (Figure 1B). The expression levels of *ICAM-1* and *EGFR* in respiratory organoids were lower than those in the human adult lung (Figure 1B). However, we confirmed that the transcripts per kilobase million (TPM) values of *ICAM-1* and *EGFR* in respiratory organoids were greater than 10 (Figure 1C).

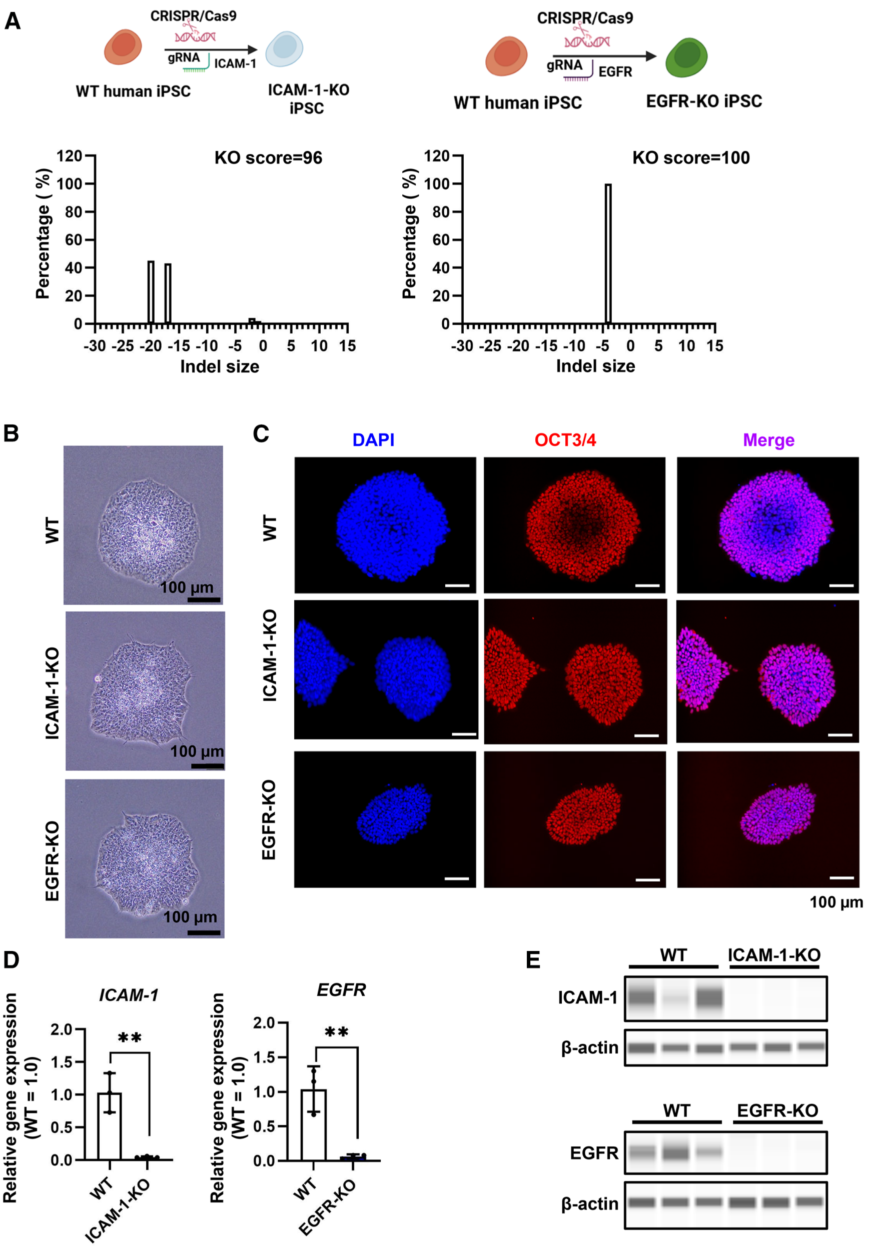

Figure 2. ICAM-1- and EGFR-KO iPSCs were established using CRISPR-Cas9 system

(A) ICAM-1-knockout (KO) and EGFR-KO iPSCs were generated using the CRISPR-Cas9 system. Bar plots represent the indel distribution for ICAM-1 and EGFR. (B) Phase images of wild-type (WT), ICAM-1-KO, and EGFR-KO iPSCs. Scale bars, 100 μm. (C) Immunofluorescence analysis of OCT3/4 (red) expression in WT, ICAM-1-KO, and EGFR-KO iPSCs. Nuclei were counterstained with DAPI (blue). Scale bars, 100 μm. (D) The expression levels of *ICAM-1* and *EGFR* in WT, ICAM-1-KO, and EGFR-KO iPSC-derived respiratory organoids were measured by RT-qPCR. Data are shown as mean ± SD (n = 3, technical replicates); two-tailed Student's t test (**$p < 0.01$). (E) The expression levels of ICAM-1, EGFR, and β-actin in WT, ICAM-1-KO, and EGFR-KO iPSC-derived respiratory organoids were measured by the capillary-based immunoassay.

These results suggest that respiratory organoids were successfully differentiated from human iPSCs.

To examine the susceptibility to RSV, we infected the respiratory organoids with RSV subtype A (RSV-A). To confirm RSV replication in respiratory organoids, we performed 50% tissue culture infectious dose ($TCID_{50}$) assays at multiple time points after infection (Figure 1D). The $TCID_{50}$ values increased over time and reached a plateau at 72 h post-infection (hpi). At 96 hpi, RSV nucleoprotein (*N*) mRNA and RSV F protein were expressed in infected respiratory organoids (Figures 1E and 1F). The expression levels of ciliated epithelial cell markers, such as *FOXJ1*, tubulin alpha-1A chain (*TUBA1A*), and multiciliate differentiation and DNA synthesis-associated cell cycle protein (*MCIDAS*) in respiratory organoids were decreased by RSV infection (Figure S1), indicating epithelial cell damage occurred because of RSV infection. These findings indicate that respiratory organoids were susceptible to RSV infection.

Generation of ICAM-1- and EGFR-KO iPSC-derived respiratory organoids

To investigate the roles of ICAM-1 and EGFR in RSV infection, we generated ICAM-1- and EGFR-KO iPSCs using the CRISPR-Cas9 system. Analysis was done using inference of CRISPR edits (ICE) (https://www.synthego.com/help/ice) showed that the KO scores for ICAM-1-KO and EGFR-KO iPSCs were nearly 100 (Figures 2A and S2A). Phase images indicated that both ICAM-1-KO and EGFR-KO iPSCs exhibit a morphology similar to that of wild-type (WT) iPSCs (Figure 2B). The expression levels of pluripotent markers (*OCT3/4*, *SOX2*, *KLF4*, *c-Myc*, and *NANOG*) in ICAM-1-KO and EGFR-KO iPSCs were similar to those in WT iPSCs (Figures S2B and S2C). In addition, immunofluorescence staining showed that both ICAM-1-KO and EGFR-KO iPSCs express OCT3/4, similar to WT iPSCs (Figure 2C). Flow cytometry analysis showed that nearly all ICAM-1-KO and EGFR-KO iPSCs were positive for TRA-1-81, a marker of undifferentiated cells (Figure S2D). These results suggest that the characteristics of human iPSCs were not affected by depleting ICAM-1 and EGFR.

ICAM-1-KO and EGFR-KO iPSCs were then differentiated into respiratory organoids. The mRNA and protein expression of ICAM-1 and

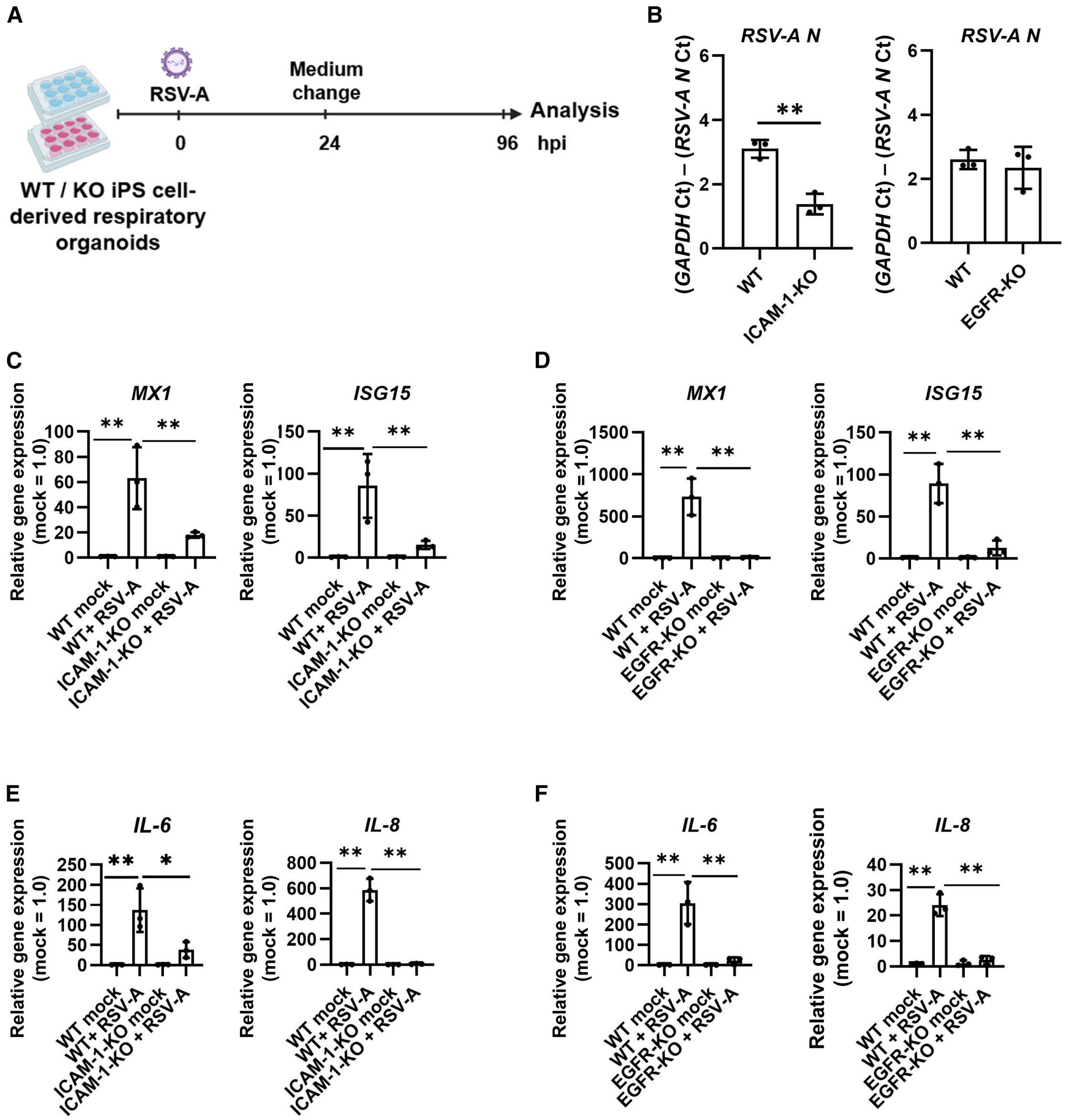

Figure 3. RSV-A infection experiment using ICAM-1 and EGFR-KO respiratory organoids

(A) WT, ICAM-1-KO, and EGFR-KO iPSC-derived respiratory organoids were infected with RSV-A at 0.1 $TCID_{50}$/cell (8×10^4 $TCID_{50}$/well) and cultured for 96 h. (B) At 96 hpi, the expression levels of *RSV N* in WT, ICAM-1-KO, and EGFR-KO respiratory organoids were measured by RT-qPCR. Data are shown as mean ± SD ($n = 3$, three infection batches); Two-tailed Student's *t* test (**$p < 0.01$). (C and D) The expression levels of *MX1* and *ISG15* in RSV-A-infected and uninfected WT, ICAM-1-KO (C), and EGFR-KO (D) respiratory organoids were measured by RT-qPCR. Data are shown as mean ± SD ($n = 3$, three infection batches); one-way analysis of variance (ANOVA) with Tukey's post hoc test (**$p < 0.01$). (E and F) The expression levels of *IL-6* and *IL-8* in RSV-A-infected and uninfected WT, ICAM-1-KO (E), and EGFR-KO (F) respiratory organoids were measured by RT-qPCR. Data are shown as mean ± SD ($n = 3$, three infection batches); one-way ANOVA with Tukey's post hoc test (*$p < 0.05$, **$p < 0.01$).

EGFR were significantly reduced in ICAM-1-KO and EGFR-KO iPSC-derived respiratory organoids, respectively (Figures 2D, 2E, and S3). The expression levels of respiratory epithelial cell markers in ICAM-1-KO and EGFR-KO iPSC-derived respiratory organoids were similar to those in WT iPSC-derived respiratory organoids (Figures S4A and S4B). These results suggest that ICAM-1-KO and EGFR-KO iPSC-derived respiratory organoids were successfully generated.

RSV infectivity was reduced in ICAM-1-KO respiratory organoids

To assess the effects of ICAM-1 and EGFR deletion on RSV infectivity, WT, ICAM-1-KO, and EGFR-KO iPSC-derived respiratory organoids were infected with RSV-A (Figure 3A). At 96 hpi, *RSV N* mRNA levels were significantly reduced in ICAM-1-KO respiratory organoids, whereas no change was observed in EGFR-KO respiratory organoids compared to WT respiratory organoids

(Figure 3B). In the infected ICAM-1-KO respiratory organoids, the expression of the genes related to innate immune and pro-inflammatory responses, such as interferon-stimulated gene 15 (*ISG15*), MX dynamin-like GTPase 1 (*MX1*), interleukin (*IL*)-*6*, and *IL-8* was significantly decreased compared with the infected WT respiratory organoids (Figures 3C and 3E). On the contrary, a similar reduction in the expression of the genes related to innate immune and pro-inflammatory responses was observed in the infected EGFR-KO respiratory organoids compared to the infected WT respiratory organoids (Figures 3D and 3F). In respiratory organoids, ICAM-1-KO and EGFR-KO caused little change in the expression levels of other RSV-related host factors (Figures S5A and S5B). These findings indicate that ICAM-1 contributed to RSV infectivity in iPSC-derived respiratory organoids.

RSV infectivity was decreased by treatment with anti-NCL antibody

To investigate the roles of NCL and IGF1R in RSV infection, we attempted to generate NCL-KO and IGF1R-KO iPSCs using the CRISPR-Cas9 system. However, both NCL-KO and IGF1R-KO-iPSCs failed to survive during the early stages of respiratory differentiation, consistent with previous reports that NCL and IGF1R are important for self-renewal and differentiation potencies of pluripotent stem cells.[21–23] As an alternative approach, we used neutralizing antibodies to inhibit NCL and IGF1R functions. WT iPSC-derived respiratory organoids were treated with anti-NCL, anti-IGF1R, or isotype control antibodies prior to the RSV infection (Figure 4A). At 96 hpi, *RSV N* gene expression was significantly reduced by anti-NCL antibodies treatment, while no change was observed in anti-IGF1R antibody-treated respiratory organoids compared to the isotype control-treated respiratory organoids (Figure 4B). The expression levels of *ISG15*, *MX1*, *IL-6*, and *IL-8* were also significantly downregulated by anti-NCL antibodies treatment (Figures 4C and 4E), while anti-IGF1R antibody treatment had no impact on the expression levels of these genes (Figures 4D and 4F). These findings suggest that NCL contributed to RSV infectivity in iPSC-derived respiratory organoids.

Dual inhibition of ICAM-1 and NCL led to a more pronounced reduction in RSV infection

To examine the combined roles of ICAM-1 and NCL in RSV infection, ICAM-1-KO iPSC-derived respiratory organoids were treated with anti-NCL antibodies and then infected with RSV (Figure 5A). At 96 hpi, the expression levels of *RSV N* and viral titers in ICAM-1-KO respiratory organoids treated with anti-NCL antibodies were significantly decreased compared to those in ICAM-1-KO respiratory organoids or WT respiratory organoids treated with isotype control antibodies (Figures 5B and 5C). In addition, we performed dose-dependent experiments using anti-ICAM-1 and anti-NCL antibodies in combination. We confirmed that higher concentrations of anti-ICAM-1 and anti-NCL antibodies resulted in lower $TCID_{50}$ values (Figure S6). Furthermore, we confirmed that the combined use of anti-ICAM-1 and anti-NCL antibodies also reduced $TCID_{50}$ values not only in respiratory organoids derived from human iPSCs (1383D6) but also in respiratory organoids derived from human embryonic stem cells (ESCs) (H9 and KhES3) (Figures S7 and S8). Even when the viral inoculum was increased 10-fold, the combined use of anti-ICAM-1 and anti-NCL antibodies reduced the $TCID_{50}$ values (Figure S9). Immunofluorescence staining showed that the number of RSV F protein-positive cells were reduced in the dual inhibition group than that of ICAM-1-KO respiratory organoids or WT respiratory organoids treated with isotype control antibodies (Figure 5D). In addition, dual inhibition of ICAM-1 and NCL significantly suppressed the expression of pro-inflammatory cytokines, *IL-6* and *IL-8*, compared to that of WT respiratory organoids treated with isotype control antibodies (Figure S10). These findings suggest that combined suppression of ICAM-1 and NCL is more effective in reducing RSV infection than inhibition of ICAM-1 alone.

DISCUSSION

In this study, we systematically examined the roles of four candidate host factors—ICAM-1, NCL, EGFR, and IGF1R—in RSV infection using a physiologically relevant human iPSC-derived respiratory organoid model. We found that suppression of ICAM-1 or NCL markedly reduced RSV infection efficiency. Notably, simultaneous inhibition of the two factors produced an enhanced antiviral effect, resulting in a greater reduction in *RSV N* gene expression and RSV F protein positivity than either inhibition alone. This enhanced effect supports the notion that these factors act at distinct but complementary stages of the viral entry process. ICAM-1 facilitates fusion by interacting with the RSV F protein on the cell surface,[14] while NCL translocates from the nucleus to the cell surface in response to RSV infection and functions as a co-receptor to promote fusion.[13,16] These distinct characteristics suggest that ICAM-1 and NCL may contribute to RSV infection at different stages of viral entry. Specifically, ICAM-1 may function as an initial receptor that supports F protein-mediated attachment or activation on the cell surface, whereas NCL may be recruited later and further enhance fusion efficiency after viral engagement. Consistent with this model, our results showing a more pronounced reduction in infection by dual inhibition support the idea that ICAM-1 and NCL may act in a sequential and cooperative manner during RSV entry. However, further mechanistic studies will be required to clarify the precise timing and functional relationship between these two host factors.

We found that simultaneous inhibition of ICAM-1 and NCL reduced RSV infection efficiency in respiratory organoids. However, in this study, we were unable to perform rescue experiments using genetically modified respiratory organoids (for example, ICAM-1-KO respiratory organoids). Because respiratory organoids contain multiple cell types, it is technically difficult to overexpress ICAM-1 in each component cell type and adjust its expression level to match that of WT respiratory organoids. Therefore, we cannot exclude the possibility that the loss of ICAM-1 may have altered the cellular composition or cellular functions of the organoids, which could affect their susceptibility to RSV infection. Thus, further studies using additional

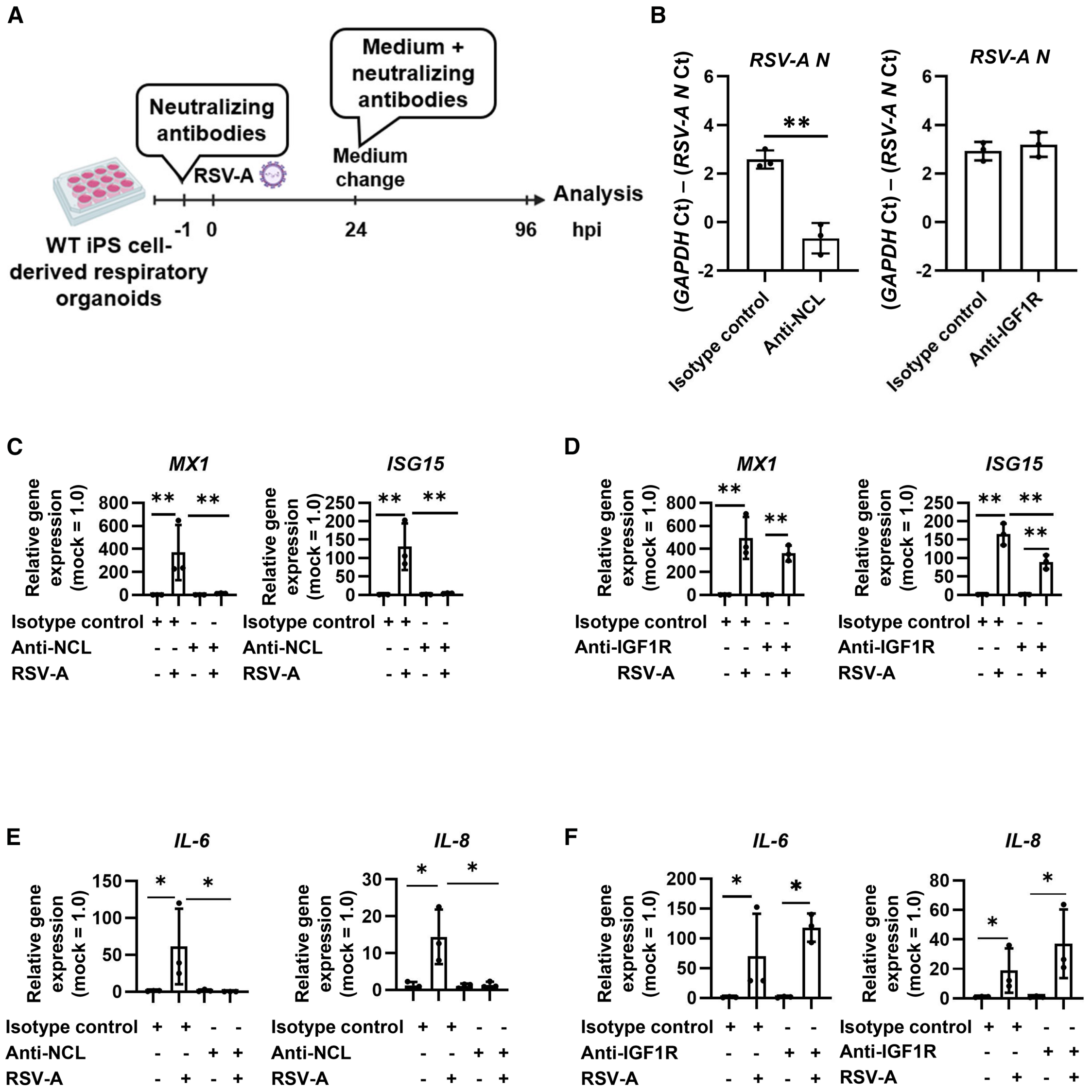

Figure 4. RSV-A infection experiment using NCL and IGF1R neutralizing antibodies

(A) WT iPSC-derived respiratory organoids were treated with anti-NCL antibodies (10 μg/mL) or anti-IGF1R antibodies (10 μg/mL), or control antibodies (10 μg/mL). After 1 h of incubation, respiratory organoids were infected with RSV-A at 0.1 $TCID_{50}$/cell (8×10^4 $TCID_{50}$/well) and cultured for 96 h. (B) At 96 hpi, the expression levels of *RSV N* in anti-NCL, anti-IGF1R, and control antibodies-treated respiratory organoids were measured by RT-qPCR. Data are shown as mean ± SD ($n = 3$, three infection batches); Two-tailed Student's *t* test (**$p < 0.01$). (C and D) The expression levels of *MX1* and *ISG15* in RSV-A-infected and uninfected respiratory organoids treated with control, anti-NCL (C), and anti-IGF1R (D) antibodies or isotype control were measured by RT-qPCR. Data are shown as mean ± SD ($n = 3$, three infection batches); one-way ANOVA with Tukey's post hoc test (**$p < 0.01$). (E, F) The expression levels of *IL-6* and *IL-8* in RSV-A-infected and uninfected respiratory organoids treated with control, anti-NCL (E), and anti-IGF1R (F) antibodies were measured by RT-qPCR. Data are shown as mean ± SD ($n = 3$, three infection batches); one-way ANOVA with Tukey's post hoc test (*$p < 0.05$, **$p < 0.01$).

experimental models, in addition to respiratory organoids, will be necessary to confirm the importance of simultaneously inhibiting ICAM-1 and NCL.

Although EGFR deletion did not alter *RSV N* mRNA expression level in our model, it markedly reduced infection-mediated upregulation of innate immune and pro-inflammatory gene expressions. Previous study suggested that EGFR is essential for toll-like receptor 3 (TLR3) signaling, and dsRNA-mediated innate immune response was attenuated in EGFR-deficient cells or EGFR inhibitor-treated cells.[24] Moreover, TLR3 is known to recognize RSV and thereby promote an antiviral response.[25] The findings of this study suggest that the involvement of EGFR in antiviral responses may be more significant than its role as a viral entry receptor.

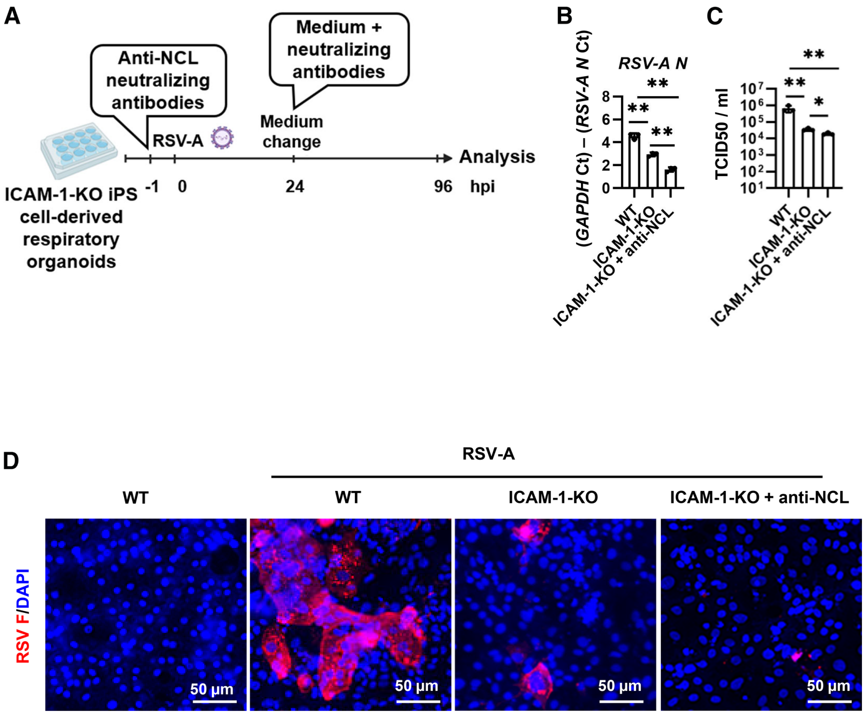

Figure 5. ICAM-1 and NCL cooperatively reduce RSV-A infection efficiency in human iPSC-derived respiratory organoids

(A) ICAM-1-KO iPSC-derived respiratory organoids were treated with anti-NCL antibodies (10 μg/mL) or an isotype control (10 μg/mL). After 1 h of incubation, respiratory organoids were infected with RSV-A at 0.1 $TCID_{50}$/cell (8×10^4 $TCID_{50}$/well) and cultured for 96 h. (B) At 96 hpi, the expression levels of *RSV N* were measured by RT-qPCR in WT, ICAM-1-KO, and anti-NCL antibody-treated ICAM-1-KO respiratory organoids. Data are shown as mean ± SD ($n = 3$, three infection batches); one-way ANOVA with Tukey's post hoc test (**$p < 0.01$). (C) At 96 hpi, the cell culture supernatants of RSV-A-infected WT, ICAM-1-KO, and anti-NCL antibody-treated ICAM-1-KO respiratory organoids were collected and $TCID_{50}$ assay was performed. Data are shown as mean ± SD ($n = 3$, three infection batches); one-way ANOVA with Tukey's post hoc test (*$p < 0.05$, **$p < 0.01$). (D) Immunofluorescence staining of RSV F protein (red) in infected WT, ICAM-1-KO, and anti-NCL–treated ICAM-1-KO respiratory organoids. Cell nuclei were counterstained with DAPI (blue). Scale bars, 50 μm.

Inhibition of IGF1R and EGFR did not significantly reduce RSV *N* expression in our respiratory organoids under our experimental conditions (Figures 3B and 4B). On the contrary, previous reports using cell lines and mouse models have found that EGFR depletion can reduce RSV infectivity.[15] Furthermore, IGF1R was identified as a critical entry receptor using primary human airway epithelial cells in air-liquid interface (ALI) culture.[16] These discrepancies likely reflect important differences among experimental systems, including species variation, cellular heterogeneity, or structural similarities to human tissues. However, it remains difficult to determine which mechanism is truly accurate without verification using human specimens. By examining RSV susceptibility in individuals carrying mutations in the four genes investigated in this study, as well as in their family members, it may be possible to obtain more detailed insights in the future.

We believe that the respiratory organoids used in this study may also be useful in future investigations of inter-individual differences in RSV susceptibility. For example, patients with asthma or chronic obstructive pulmonary disease (COPD) exhibit elevated ICAM-1 and EGFR expression[26–28] and are at increased risk of severe RSV infection.[29] Future work using patient-specific iPSC-derived respiratory organoids could directly evaluate how disease-associated states shape RSV entry, immune activation, and severity. This platform, therefore, represents a promising tool for evaluating patient-specific viral-host interaction and the discovery of new therapeutic interventions.

Despite the strong reduction in viral replication by dual ICAM-1 and NCL inhibition, infection was not completely abolished. This indicates that additional host factors may contribute to RSV entry. Although we focused on F-protein-interacting factors, prior studies have implicated several G-protein-associated molecules, like CX3CR1,[30] annexin II,[31] and surfactant protein-A,[32] as potential attachment or co-receptors. Therefore, a more comprehensive screening that includes these factors involved in viral entry will be necessary. Another limitation is that NCL and IGF1R were important for the differentiation of respiratory organoids from human iPSCs, preventing the use of NCL- and IGF1R-KO cell lines for analysis. Therefore, in this study, these two host factors were examined using neutralizing antibodies. Neutralizing antibodies are unlikely to achieve complete inhibition. To precisely elucidate the roles of NCL and IGF1R, it is necessary to establish respiratory organoids with conditional KO of these factors and evaluate their functions.

MATERIALS AND METHODS

Human ESCs/iPSCs

The human iPSC line, 1383D6 (provided by Dr. Masato Nakagawa, Kyoto University), and human ESC lines, H9 (WA09, WiCell Research Institute) and KhES3 (Kyoto University), were maintained on 0.5 μg/cm^2 recombinant human laminin 511 E8 fragments (iMatrix-511, Cat# 892012, Nippi) with StemFit AK02N medium (Cat# RCAK02N, Ajinomoto Healthy Supply). Cell passage was performed every 6 days. For cell passaging, human ESC/iPSC colonies were treated with TrypLE Select Enzyme (Cat# 12563029, Thermo Fisher Scientific) for 10 min at 37°C and seeded with StemFit AK02N medium containing 5 μM Y-27632 (Cat# 034–24024, FUJIFILM Wako Pure Chemical). Human ESCs were used following the guidelines for derivation and utilization of human embryonic stem cells of the

Ministry of Education, Culture, Sports, Science, and Technology of Japan, and the study was approved by an independent ethics committee. We used the human ESC lines, H9 and KhES3, only for the $TCID_{50}$ assay shown in Figures S7 and S8, respectively, while all other experiments were performed using the human iPSC line 1383D6. The phase images shown in Figure 2B were obtained with a CKX53 microscope (Evident).

Respiratory organoids

To start the differentiation, human ESC/iPSC colonies were treated with TrypLE Select Enzyme (Cat# 12563029, Thermo Fisher Scientific) for 10 min at 37°C. After centrifugation, cells were seeded onto Matrigel growth factor reduced basement membrane (Cat# 354230, Corning Life Sciences)-coated cell culture plates (2.0×10^5 cells/4 cm^2) and cultured for 2 days. The differentiation of the respiratory organoids was performed in serum-free differentiation (SFD) medium, composed of DMEM/F12 (3:1) (Cat# 044–29765, FUJIFILM Wako Pure Chemical and Cat# 11320033, Thermo Fisher Scientific) supplemented with N2 (Cat# 141–08941, FUJIFILM Wako Pure Chemical), B-27 supplement minus vitamin A (Cat# 12587001, Thermo Fisher Scientific), ascorbic acid (50 μg/mL, Cat# ST-72132, STEMCELL Technologies), 1× GlutaMAX (Cat# 35050–061, Thermo Fisher Scientific), 0.5 mmol/mL monothioglycerol (Cat# 195–15791, FUJIFILM Wako Pure Chemical), 0.05% bovine serum albumin (Cat# 820024, Sigma-Aldrich), and 1× penicillin/streptomycin. During days 0–1 of differentiation, cells were cultured with SFD medium supplemented with 10 μM Y-27632 (Cat# 034–24024, FUJIFILM Wako Pure Chemical) and 100 ng/mL recombinant activin A (Cat# 338-AC-01 M, R&D Systems). During days 1–3 of differentiation, cells were cultured with SFD medium supplemented with 10 μM Y-27632 (Cat# 034–24024, FUJIFILM Wako Pure Chemical), 100 ng/mL recombinant activin A (Cat# 338-AC-01 M, R&D Systems) and 1% fetal bovine serum (FBS). During days 3–5, cells were cultured in SFD medium supplemented with 1.5 μM dorsomorphin dihydrochloride (Cat# 047–33763, FUJIFILM Wako Pure Chemical) and 10 μM SB431542 (Cat# 037–24293, FUJIFILM Wako Pure Chemical) for 24 h, followed by SFD medium supplemented with 10 μM SB431542 and 1 μM IWP2 (Cat# 04–0034, Stemolecule) for an additional 24 h. During days 5–12, cells were cultured with SFD medium supplemented with 3 μM CHIR99021 (Cat# 034–23103, FUJIFILM Wako Pure Chemical), 10 ng/mL human FGF10 (Cat# AF-100-26, PeproTech), 10 ng/mL human FGF7 (Cat# AF-100-19, PeproTech), 10 ng/mL human BMP4 (Cat# 120-05ET, PeproTech), 20 ng/mL human epidermal growth factor (EGF) (Cat# AF-100-15, PeproTech), and 50 nM all-*trans* retinoic acid (ATRA, Cat# R2625, Sigma-Aldrich). On day 12, cells were dissociated and embedded in the Matrigel growth factor reduced basement membrane to generate organoids. During days 12–20, organoids were cultured in SFD medium containing 3 μM CHIR99021, 10 ng/mL human FGF10, 10 ng/mL human FGF7, 10 ng/mL human BMP4, and 50 nM ATRA. On day 20 of differentiation, organoids were recovered from the Matrigel, and the resulting suspension of organoids (small free-floating clumps) was seeded onto Matrigel-coated cell culture plates. During days 20–30 of differentiation, organoids were cultured in SFD medium containing 50 nM dexamethasone (Cat# S1322, Selleck Chemicals), 0.1 mM 8-bromo-cAMP (Cat# 1140/50, Tocris Bioscience), and 0.1 mM 3-isobutyl-1-methylxanthine, (IBMX, Cat# 095–03413, FUJIFILM Wako Pure Chemical).

RSV

The RSV isolate RSV/Sendai/28–30 (subgroup A) was used in this study (Global Initiative on Sharing All Influenza Data [GISAID] accession number: EPI_ISL_19696283). The virus was kindly provided by Dr. Hidekazu Nishimura (Sendai Medical Center). RSV was propagated in HEp-2 cells (Cat# CCL-23, ATCC) and stored at −80°C. HEp-2 cells were cultured in Eagle's minimum essential medium (EMEM) (Cat# 051–07615, FUJIFILM Wako Pure Chemical) supplemented with 10% FBS and 1% penicillin/streptomycin.

Viral titration

Viral titers were determined by a $TCID_{50}$ assay. HEp-2 cells were seeded into 96-well plates (Cat# 167008, Thermo Fisher Scientific). Then, samples were serially diluted 10-fold from 10^{-1} to 10^{-8} in culture medium, added to the cells, and incubated at 37°C for 96 h. Cytopathic effects were assessed under a microscope. $TCID_{50}$/mL values were calculated using the Reed-Muench method.

Generation of ICAM-1- or EGFR-KO iPSCs

The ICAM-1 and EGFR loci were targeted using CRISPR-Cas9 ribonucleoprotein (RNP) complexes. Human iPSCs (1.0×10^6 cells) were electroporated with CRISPR-Cas9 RNP complexes consisting of 60 pmol Alt-R S. p. Cas9 Nuclease v.3 (Cat# 108303556, IDT) and 120 pmol gRNA. Electroporation was performed using the ExPERT ATx electroporation system (MaxCyte). After electroporation, cells were seeded onto iMatrix-511-coated dishes and cultured in medium containing 10 μM Y-27632. Ten days after electroporation, individual colonies were picked and transferred to iMatrix-511-coated 12-well plates. When the wells reached near confluence, genotyping was performed using specific primers to confirm correct targeting of each clone. The gRNA sequences and primer sequences used for genotyping are listed in Table S1. The frequency of insertions and deletions (indels) was analyzed using the ICE tool.

RT-qPCR

Total RNA was isolated from respiratory organoids using ISOGENE (Cat# 319–90211, NIPPON GENE). Human adult lung total RNA was purchased from Thermo Fisher Scientific (Cat# QS0618) and used as a control. cDNA was synthesized from 500 ng of total RNA using the SuperScript VILO cDNA Synthesis Kit (Cat# 11754250, Thermo Fisher Scientific). Quantitative real-time PCR was performed using SYBR Green PCR Master Mix (Cat# 4385614, Thermo Fisher Scientific) on a QuantStudio 1 or QuantStudio 3 real-time PCR system. Relative mRNA expression levels were calculated using the $2^{-\Delta\Delta Ct}$ method and normalized to the housekeeping gene glyceraldehyde 3-phosphate dehydrogenase (*GAPDH*). Primer sequences are listed in Table S2.

RNA-seq

RNA sequencing (RNA-seq) analysis in human iPSC-derived respiratory organoids was performed in our previous study[18] Gene Expression Omnibus (GEO accession number: GSE263272). TPM values showed in Figure 1C.

Flow cytometry

Single-cell suspensions of human iPSCs were prepared and then incubated with primary antibodies, followed by secondary antibodies. Primary and secondary antibodies are summarized in Table S3. Flow cytometric analysis was performed using a MACSQuant Analyzer (Miltenyi Biotec), and data were analyzed with FlowJo software (BD Life Sciences).

Capillary-based immunoassay

Respiratory organoids were lysed in Radio-Immunoprecipitation Assay (RIPA) buffer (Cat# 89900, Thermo Fisher Scientific) supplemented with a protease inhibitor cocktail (Cat# P8340, Sigma-Aldrich). After centrifugation, supernatants were collected. Protein expression was analyzed using the Jess Simple Western system (ProteinSimple) with the 12–230 kDa Separation Module (Cat# SM-W001, ProteinSimple) according to the manufacturer's instructions. The antibodies used are listed in Table S3. Data were analyzed and visualized using Compass for Simple Western software (ProteinSimple).

Immunofluorescence staining

For immunofluorescence staining, cells were fixed with 4% paraformaldehyde in PBS. Respiratory organoids were embedded in Tissue-Tek O.C.T. Compound (Sakura Finetek, Japan) and sectioned at approximately 5 μm. Antigen retrieval was performed using 0.1% Tween 20 in tris-buffered saline (TBS) (pH 7.4) (Cat# 12750–81, Nacalai Tesque). Sections of respiratory organoids were incubated with Blocking One (Cat# 06349–64, Nacalai Tesque) for 10 min at room temperature to prevent nonspecific binding. Samples were incubated overnight at 4°C with a primary antibody against RSV (Cat# AB43812, Abcam). The following day, cells were washed three times with 1 × PBS (Cat# 14249–24, Nacalai Tesque). Alexa Fluor 594-conjugated secondary antibody incubation was performed at room temperature for 45 min, followed by three washes with PBS for 5 min each. Sections were mounted with ProLong Glass Antifade Mountant with NucBlue Stain (Cat# P36985, Thermo Fisher Scientific) and analyzed using a BZ-X700 fluorescence microscope (Keyence Corporation).

Neutralizing antibodies

Respiratory organoids were cultured in the medium containing anti-NCL antibodies (Cat# SC-8031, Santa Cruz Biotechnology), anti-IGF1R antibodies (Cat# AF-305-NA, R&D Systems), anti-ICAM-1 antibodies (Cat# RSD-AF720, R&D Systems), or IgG control (Cat# AB-108-C, R&D Systems) for 1 h. Cells were then infected with RSV. At 96 hpi, total RNA collected, and then RT-qPCR was performed (see also RT-qPCR section).

Statistical analyses

Statistical significance was evaluated using an unpaired two-tailed Student's *t* test or one-way analysis of variance (ANOVA) followed by Tukey's post hoc test, as appropriate. Statistical analyses were performed using GraphPad Prism 9. Data are representative of three independent experiments. Additional details are provided in the figure legends.

DATA AND CODE AVAILABILITY

Bulk RNA-seq data used in this study was previously submitted to the GEO under accession number GSE263272. Detailed information including genomic sequence of isolated RSV (RSV/Sendai/28–30 (subgroup A)) used in this study was previously submitted to GISAID under accession number EPI_ISL_19696283.

ACKNOWLEDGMENTS

We thank Dr. Hidekazu Nishimura (Sendai Medical Center) for preparing RSV isolates, Dr. Peter Gee and Dr. Masahisa Ohishi (MaxCyte) for their support with CRISPR-Cas9 experiments, Ms. Natsumi Mimura and Naoko Yasuhara (Institute of Science Tokyo), and Shiho Morimoto (Kyoto University) for technical assistance. Figures 2A, 3A, 4A, and 5A were created with BioRender.com.

This research was supported by the Japan Agency for Medical Research and Development, Japan (AMED) (JP21gm1610005 and JP23jf0126002), JSPS Core-to-Core Program, Japan (A. Advanced Research Networks and JPJSCCA20240006), JSPS KAKENHI, Japan (25K11431), Multilayered Stress Diseases, Japan (JPMXP1323015483), Science Tokyo, the iPS Cell Research Fund, and JST SPRING, Japan (JPMJSP2110).

AUTHOR CONTRIBUTIONS

A.K. conducted all experiments and wrote the manuscript; R.H. conducted RSV experiments and reviewed the manuscript; Y.K. conducted RSV experiments; Y.N. conducted CRISPR-Cas9 experiments; A.S. conducted cell culture and RT-qPCR analyses; S.G. performed administrative support; M.T. conducted RSV experiments; K.T. designed research, reviewed the manuscript, and acquired funding.

DECLARATION OF INTERESTS

The authors declare no competing financial interests.

SUPPLEMENTAL INFORMATION

Supplemental information can be found online at https://doi.org/10.1016/j.omtn.2026.102932.

REFERENCES

1. Wang, X., Li, Y., Shi, T., Bont, L.J., Chu, H.Y., Zar, H.J., Wahi-Singh, B., Ma, Y., Cong, B., Sharland, E., et al. (2024). Global disease burden of and risk factors for acute lower respiratory infections caused by respiratory syncytial virus in preterm infants and young children in 2019: a systematic review and meta-analysis of aggregated and individual participant data. Lancet *403*, 1241–1253.
2. McLaughlin, J.M., Khan, F., Begier, E., Swerdlow, D.L., Jodar, L., and Falsey, A.R. (2022). Rates of Medically Attended RSV Among US Adults: A Systematic Review and Meta-analysis. Open Forum Infect. Dis. *9*, ofac300.
3. Principi, N., Autore, G., Ramundo, G., and Esposito, S. (2023). Epidemiology of Respiratory Infections during the COVID-19 Pandemic. Viruses *15*, 1160.
4. Chuang, Y.C., Lin, K.P., Wang, L.A., Yeh, T.K., and Liu, P.Y. (2023). The Impact of the COVID-19 Pandemic on Respiratory Syncytial Virus Infection: A Narrative Review. Infect. Drug Resist. *16*, 661–675.
5. Surie, D., Yuengling, K.A., DeCuir, J., Zhu, Y., Gaglani, M., Ginde, A.A., Talbot, H.K., Casey, J.D., Mohr, N.M., Ghamande, S., et al. (2023). Disease Severity of Respiratory Syncytial Virus Compared with COVID-19 and Influenza Among Hospitalized Adults Aged ≥60 Years - IVY Network, 20 U.S. States, February 2022-May 2023. MMWR Morb. Mortal. Wkly. Rep. *72*, 1083–1088.

6. Battles, M.B., and McLellan, J.S. (2019). Respiratory syncytial virus entry and how to block it. Nat. Rev. Microbiol. *17*, 233–245.

7. Vidal Valero, M. (2023). 'A good day': FDA approves world's first RSV vaccine. Nature *617*, 234–235.

8. Harris, E. (2023). FDA Approves Maternal RSV Vaccine. JAMA *330*, 1029.

9. Mullard, A. (2024). FDA approves mRNA-based RSV vaccine. Nat. Rev. Drug Discov. *23*, 487.

10. Anderer, S. (2025). FDA Approves New RSV Monoclonal Antibody to Protect Infants. JAMA *334*, 383.

11. Harris, E. (2023). FDA Approves RSV Monoclonal Antibody for Infants and Young Children. JAMA *330*, 586.

12. Langedijk, A.C., and Bont, L.J. (2023). Respiratory syncytial virus infection and novel interventions. Nat. Rev. Microbiol. *21*, 734–749.

13. Tayyari, F., Marchant, D., Moraes, T.J., Duan, W., Mastrangelo, P., and Hegele, R.G. (2011). Identification of nucleolin as a cellular receptor for human respiratory syncytial virus. Nat. Med. *17*, 1132–1135.

14. Behera, A.K., Matsuse, H., Kumar, M., Kong, X., Lockey, R.F., and Mohapatra, S.S. (2001). Blocking intercellular adhesion molecule-1 on human epithelial cells decreases respiratory syncytial virus infection. Biochem. Biophys. Res. Commun. *280*, 188–195.

15. Currier, M.G., Lee, S., Stobart, C.C., Hotard, A.L., Villenave, R., Meng, J., Pretto, C.D., Shields, M.D., Nguyen, M.T., Todd, S.O., et al. (2016). EGFR Interacts with the Fusion Protein of Respiratory Syncytial Virus Strain 2-20 and Mediates Infection and Mucin Expression. PLoS Pathog. *12*, e1005622.

16. Griffiths, C.D., Bilawchuk, L.M., McDonough, J.E., Jamieson, K.C., Elawar, F., Cen, Y., Duan, W., Lin, C., Song, H., Casanova, J.L., et al. (2020). IGF1R is an entry receptor for respiratory syncytial virus. Nature *583*, 615–619.

17. Altamirano-Lagos, M.J., Díaz, F.E., Mansilla, M.A., Rivera-Pérez, D., Soto, D., McGill, J.L., Vasquez, A.E., and Kalergis, A.M. (2019). Current Animal Models for Understanding the Pathology Caused by the Respiratory Syncytial Virus. Front. Microbiol. *10*, 873.

18. Hashimoto, R., Watanabe, Y., Keshta, A., Sugiyama, M., Kitai, Y., Hirabayashi, A., Yasuhara, N., Morimoto, S., Sakamoto, A., Matsumura, Y., et al. (2025). Human iPS cell-derived respiratory organoids as a model for respiratory syncytial virus infection. Life Sci. Alliance *8*, e202402837.

19. Tamura, T., Mizuma, K., Nasser, H., Deguchi, S., Padilla-Blanco, M., Oda, Y., Uriu, K., Tolentino, J.E.M., Tsujino, S., Suzuki, R., et al. (2024). Virological characteristics of the SARS-CoV-2 BA.2.86 variant. Cell Host Microbe *32*, 170–180.e12.

20. Hashimoto, R., Tamura, T., Watanabe, Y., Sakamoto, A., Yasuhara, N., Ito, H., Nakano, M., Fuse, H., Ohta, A., Noda, T., et al. (2023). Evaluation of Broad Anti-Coronavirus Activity of Autophagy-Related Compounds Using Human Airway Organoids. Mol. Pharm. *20*, 2276–2287.

21. Okawa, E.R., Gupta, M.K., Kahraman, S., Goli, P., Sakaguchi, M., Hu, J., Duan, K., Slipp, B., Lennerz, J.K., and Kulkarni, R.N. (2021). Essential roles of insulin and IGF-1 receptors during embryonic lineage development. Mol. Metab. *47*, 101164.

22. Wamaitha, S.E., Grybel, K.J., Alanis-Lobato, G., Gerri, C., Ogushi, S., McCarthy, A., Mahadevaiah, S.K., Healy, L., Lea, R.A., Molina-Arcas, M., et al. (2020). IGF1-mediated human embryonic stem cell self-renewal recapitulates the embryonic niche. Nat. Commun. *11*, 764.

23. Yang, A., Shi, G., Zhou, C., Lu, R., Li, H., Sun, L., and Jin, Y. (2011). Nucleolin maintains embryonic stem cell self-renewal by suppression of p53 protein-dependent pathway. J. Biol. Chem. *286*, 43370–43382.

24. Yamashita, M., Chattopadhyay, S., Fensterl, V., Saikia, P., Wetzel, J.L., and Sen, G.C. (2012). Epidermal growth factor receptor is essential for Toll-like receptor 3 signaling. Sci. Signal. *5*, ra50.

25. Rudd, B.D., Smit, J.J., Flavell, R.A., Alexopoulou, L., Schaller, M.A., Gruber, A., Berlin, A.A., and Lukacs, N.W. (2006). Deletion of TLR3 alters the pulmonary immune environment and mucus production during respiratory syncytial virus infection. J. Immunol. *176*, 1937–1942.

26. Shukla, S.D., Mahmood, M.Q., Weston, S., Latham, R., Muller, H.K., Sohal, S.S., and Walters, E.H. (2017). The main rhinovirus respiratory tract adhesion site (ICAM-1) is upregulated in smokers and patients with chronic airflow limitation (CAL). Respir. Res. *18*, 6.

27. Kim, S., and Nadel, J.A. (2009). Fibrinogen binding to ICAM-1 promotes EGFR-dependent mucin production in human airway epithelial cells. Am. J. Physiol. Lung Cell. Mol. Physiol. *297*, L174–L183.

28. Liu, Y., Li, P., Jiang, T., Li, Y., Wang, Y., and Cheng, Z. (2023). Epidermal growth factor receptor in asthma: A promising therapeutic target? Respir. Med. *207*, 107117.

29. Landi, S.N., Garofalo, D.C., Reimbaeva, M., Scott, A.M., Jiang, L., Cappell, K., Lewandowski, D., Bonafede, M., Brzozowski, K., Drebert, Z., et al. (2024). Hospitalization Following Outpatient Diagnosis of Respiratory Syncytial Virus in Adults. JAMA Netw. Open *7*, e2446010.

30. Johnson, S.M., McNally, B.A., Ioannidis, I., Flano, E., Teng, M.N., Oomens, A.G., Walsh, E.E., and Peeples, M.E. (2015). Respiratory Syncytial Virus Uses CX3CR1 as a Receptor on Primary Human Airway Epithelial Cultures. PLoS Pathog. *11*, e1005318.

31. Malhotra, R., Ward, M., Bright, H., Priest, R., Foster, M.R., Hurle, M., Blair, E., and Bird, M. (2003). Isolation and characterisation of potential respiratory syncytial virus receptor(s) on epithelial cells. Microbes Infect. *5*, 123–133.

32. Hickling, T.P., Malhotra, R., Bright, H., McDowell, W., Blair, E.D., and Sim, R.B. (2000). Lung surfactant protein A provides a route of entry for respiratory syncytial virus into host cells. Viral Immunol. *13*, 125–135.

OMTN, Volume *37*

Supplemental information

Dual inhibition of intercellular adhesion molecule-1 and nucleolin reduces RSV infection efficiency in human respiratory organoids

Abeer Keshta, Rina Hashimoto, Yuki Kitai, Yoshitaka Nakata, Ayaka Sakamoto, Shimpei Gotoh, Makoto Takeda, and Kazuo Takayama

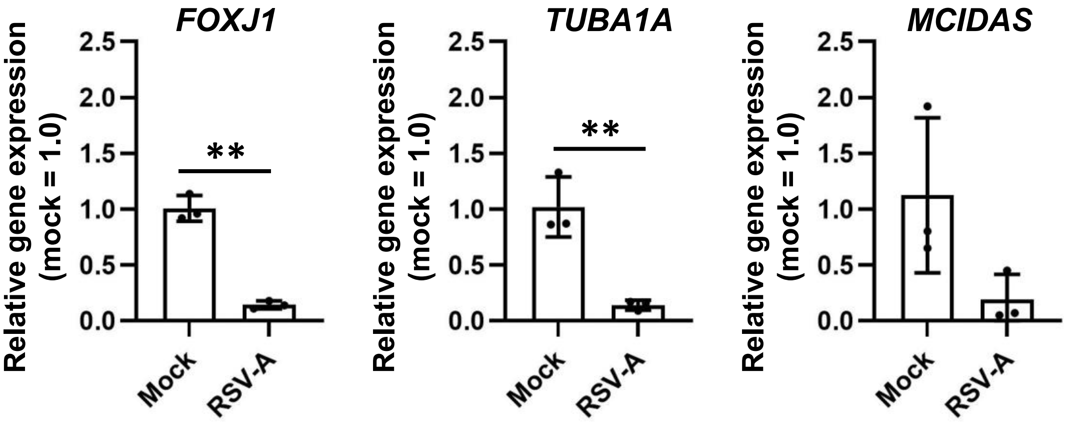

Figure S1. The gene expression levels of airway epithelial cell markers decreased in RSV-A-infected respiratory organoids, related to Figure 1. Human iPSC-derived respiratory organoids were infected with RSV-A at 0.1 TCID/cell (8 × 10^4 TCID/well) and cultured for 96 h. The expression levels of *FOXJ1*, *TUBA1A*, *and MCIDAS* in respiratory organoids were measured by RT-qPCR (mock = 1.0). Data are shown as mean ± SD (*n*=3, three infection batches); Two-tailed Student's *t*-test (***p* < 0.01).

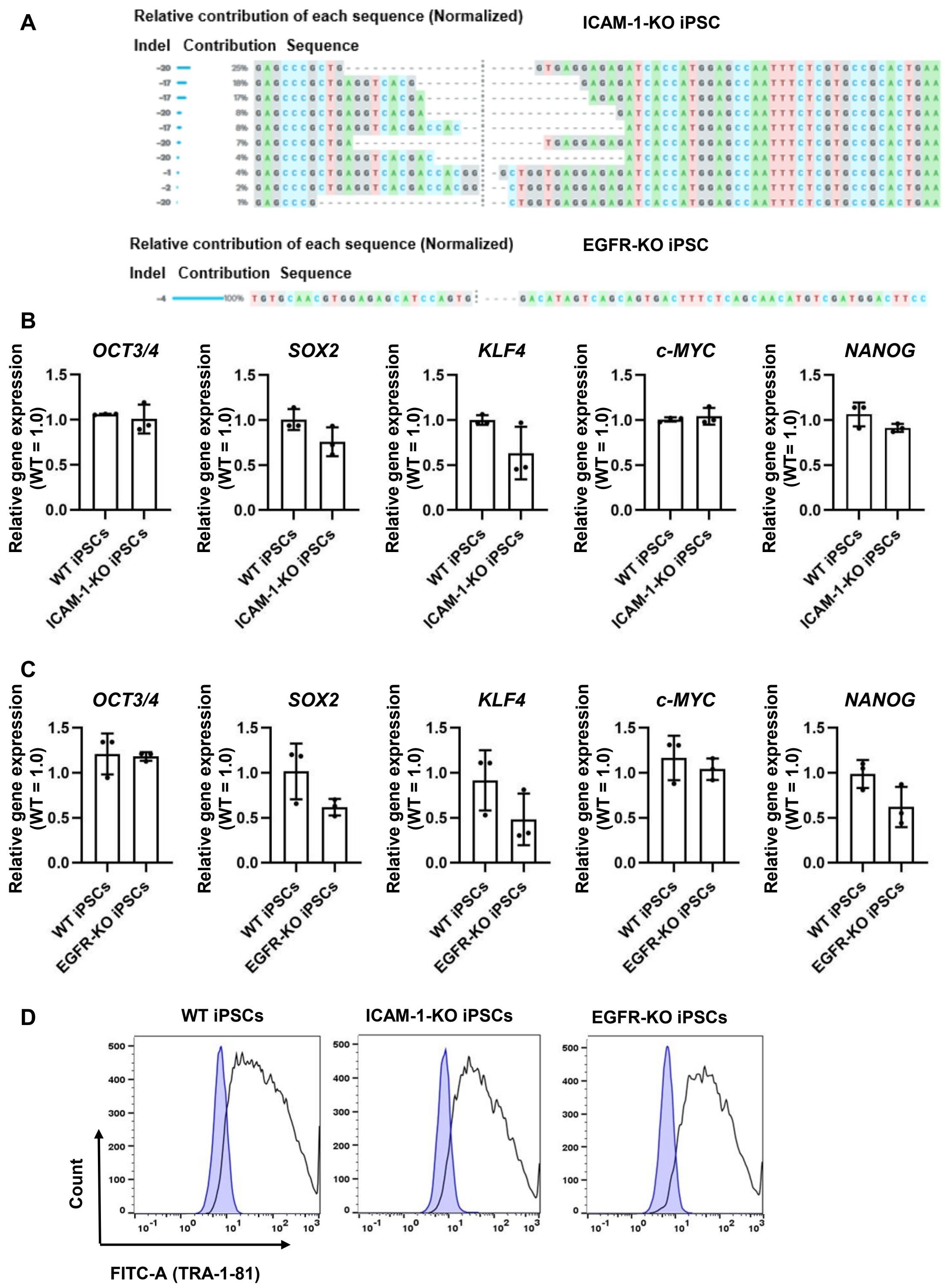

Figure S2. The genome editing efficiency and the expression level of pluripotent markers in ICAM-1- and EGFR-KO iPSCs, related to Figure 2.

(**A**) The indel contribution in each sequence of ICAM-1- or EGFR-KO iPSCs was determined using ICE analysis. (**B**) The gene expression levels of pluripotent

markers, *OCT3/4*, *SOX2*, *KLF4*, *c-MYC*, and *NANOG* in ICAM-1-KO iPSCs were examined by RT-qPCR. Data are shown as mean ± SD (*n*=3, technical replicates). (**C**) The gene expression levels of *OCT3/4*, *SOX2*, *KLF4*, *c-MYC*, and *NANOG* in EGFR-KO iPSCs. Data are shown as mean ± SD (*n*=3, technical replicates). (**D**) The histograms of human iPSCs stained with TRA-1-81 antibody (white) or unstained controls (blue) were obtained by flow cytometry.

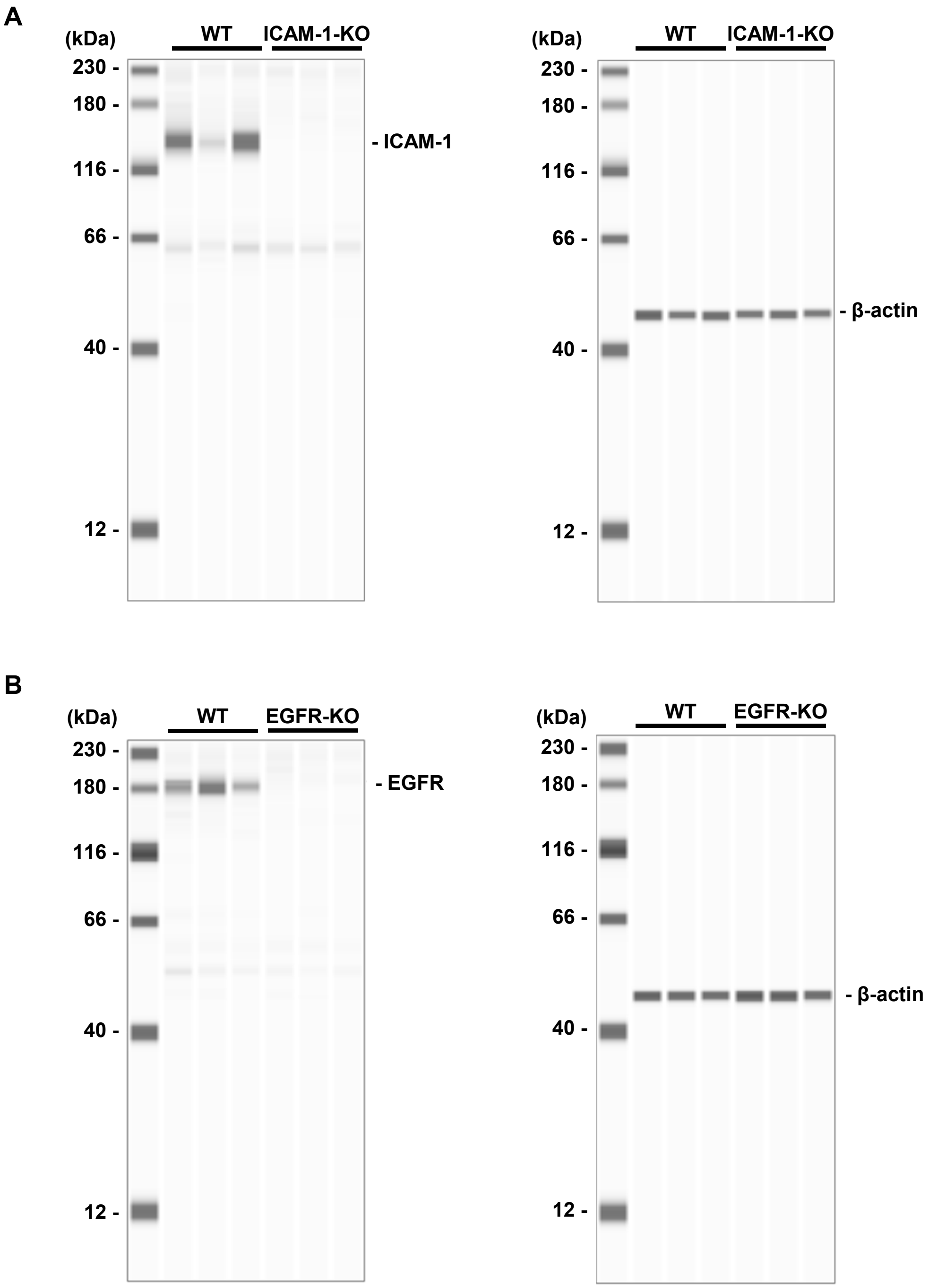

Figure S3. Protein expression analysis in ICAM-1-KO and EGFR-KO respiratory organoids, related to Figure 2.

Full gel images of capillary-based immunoassay of **Figure 2E**. (**A**) ICAM-1 and β-actin expression in ICAM-1-KO respiratory organoids. (**B**) EGFR and β-actin expression in EGFR-KO respiratory organoids.

A

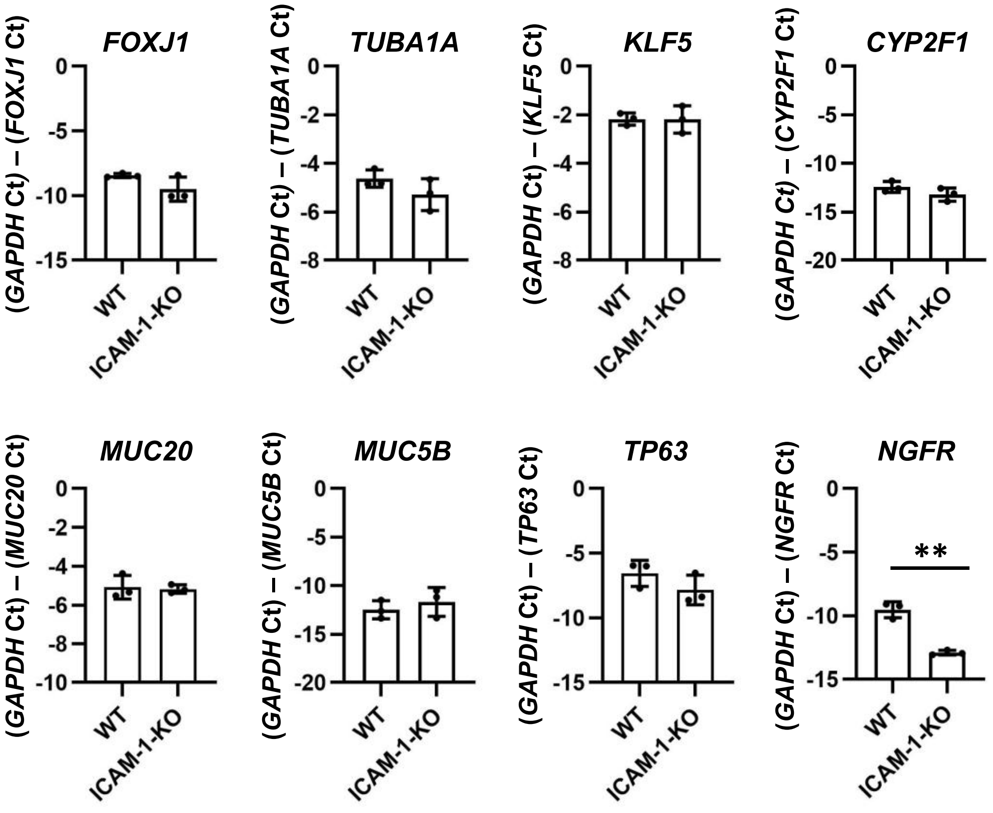
FOXJ1
(GAPDH Ct) – (FOXJ1 Ct)
TUBA1A
(GAPDH Ct) – (TUBA1A Ct)
KLF5
(GAPDH Ct) – (KLF5 Ct)
CYP2F1
(GAPDH Ct) – (CYP2F1 Ct)
MUC20
(GAPDH Ct) – (MUC20 Ct)
MUC5B
(GAPDH Ct) – (MUC5B Ct)
TP63
(GAPDH Ct) – (TP63 Ct)
NGFR
(GAPDH Ct) – (NGFR Ct)
**
WT
ICAM-1-KO

B

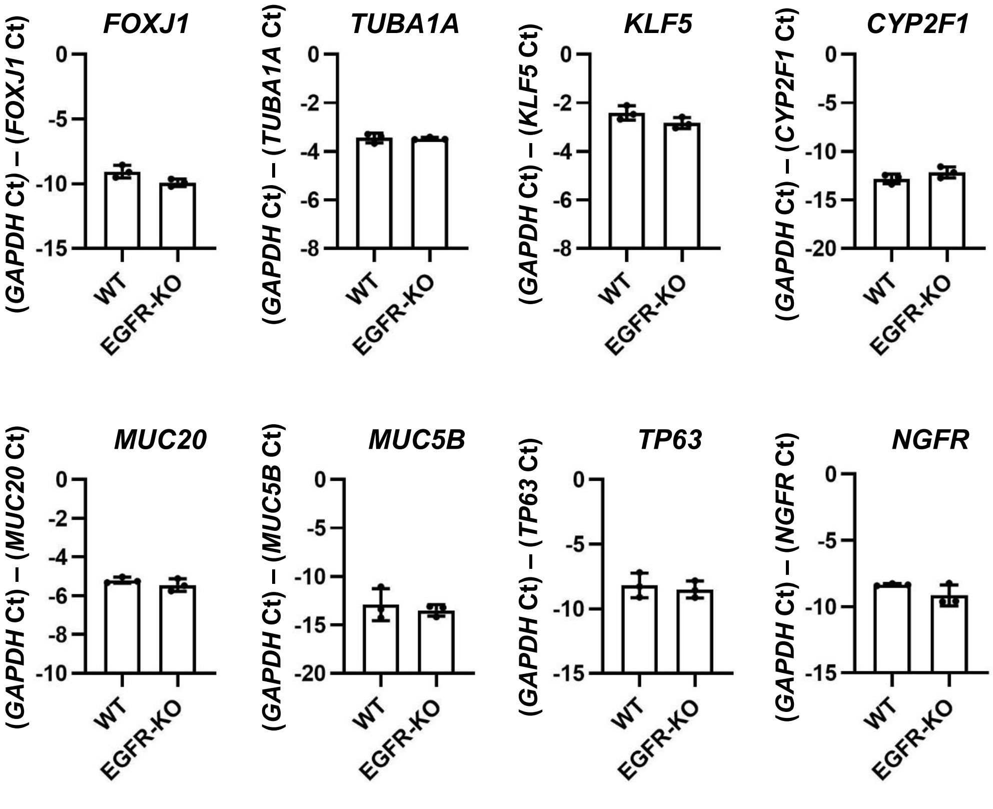
FOXJ1
(GAPDH Ct) – (FOXJ1 Ct)
TUBA1A
(GAPDH Ct) – (TUBA1A Ct)
KLF5
(GAPDH Ct) – (KLF5 Ct)
CYP2F1
(GAPDH Ct) – (CYP2F1 Ct)
MUC20
(GAPDH Ct) – (MUC20 Ct)
MUC5B
(GAPDH Ct) – (MUC5B Ct)
TP63
(GAPDH Ct) – (TP63 Ct)
NGFR
(GAPDH Ct) – (NGFR Ct)
WT
EGFR-KO

Figure S4. The gene expression levels of airway epithelial cell markers between WT and KO iPSC-derived respiratory organoids, related to Figure 2.

(**A**) The gene expression levels of ciliated cell markers (*FOXJ1*, *TUBA1A*), goblet cell markers (*MUC20*, *MUC5B*), club cell markers (*KLF5*, *CYP2F1*), and basal cell markers (*TP63*, *NGFR*) in WT and ICAM-1-KO iPSC-derived respiratory organoids were examined by RT-qPCR. Data are shown as mean ± SD (n=3, technical replicates); Two-tailed Student's t-test (**$p < 0.01$). (**B**) The gene expression levels of ciliated cell markers, goblet cell markers, club cell markers, and basal cell markers in WT and EGFR-KO iPSC-derived respiratory organoids were examined by RT-qPCR. Data are shown as mean ± SD (n=3, technical replicates).

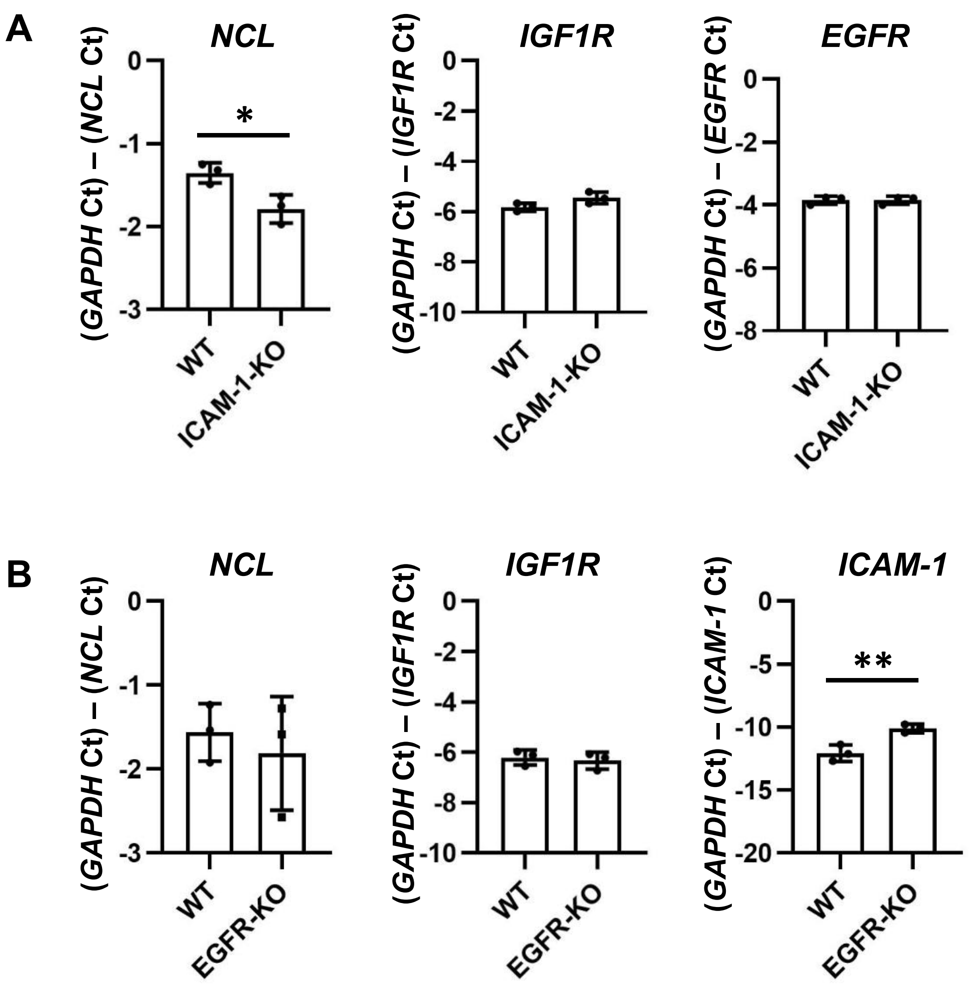

Figure S5. The gene expression levels of RSV F-associated genes between WT and KO iPSC-derived respiratory organoids, related to Figure 2.

(A) The gene expression levels of *NCL*, *IGF1R,* and *EGFR* in WT and ICAM-1-KO iPSC-derived respiratory organoids were examined by PT-qPCR. Data are shown as mean ± SD (n=3, technical replicates); Two-tailed Student's t-test (*$p < 0.05$). (**B**) The gene expression levels of *NCL*, *IGF1R,* and *ICAM-1* in WT and EGFR-KO iPSC-derived respiratory organoids were examined by RT-qPCR. Data are shown as mean ± SD (n=3); Two-tailed Student's t-test (**$p < 0.01$, technical replicates).

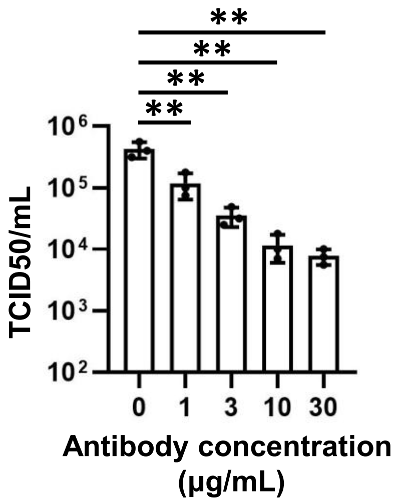

Figure S6. RSV-A infection efficiency decreased in a concentration-dependent manner with increasing levels of neutralizing antibodies, related to Figure 5.

WT iPSC-derived respiratory organoids were treated simultaneously with an anti-NCL antibody and an anti-ICAM-1 antibody. The concentration of each antibody was 0, 1, 3, 10, or 30 μg/mL. After 1 h of incubation, respiratory organoids were infected with RSV-A at 0.1 TCID/cell (8×10^4 TCID/well) and cultured for 96 h. At 96 hpi, the cell culture supernatants of RSV-A-infected WT or antibody-treated respiratory organoids were collected and $TCID_{50}$ assay was performed. Data are shown as mean ± SD (*n*=3, three infection batches); One-way ANOVA with Tukey's post hoc test (**$p < 0.01$).

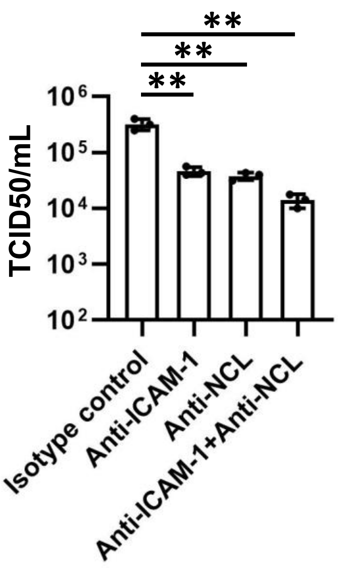

Figure S7. NCL and ICAM-1 also play important roles in RSV-A infection in human ESC-derived respiratory organoids, related to Figure 5.

WT ESC (H9)-derived respiratory organoids were treated with anti-NCL antibodies (10 μg/mL), anti-ICAM-1 antibodies (10 μg/mL), or isotype control (10 μg/mL). After 1 h of incubation, respiratory organoids were infected with RSV-A at 0.1 TCID/cell (8×10^4 TCID/well) and cultured for 96 h. At 96 hpi, the cell culture supernatants of RSV-A-infected WT or antibody-treated respiratory organoids were collected and $TCID_{50}$ assay was performed. Data are shown as mean ± SD (n=3, three infection batches); One-way ANOVA with Tukey's post hoc test (**p < 0.01).

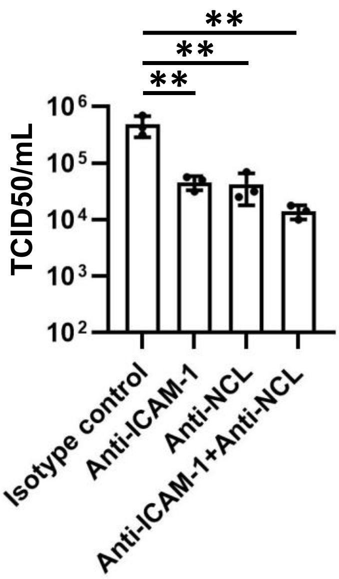

Figure S8. NCL and ICAM-1 also play important roles in RSV-A infection in human ESC-derived respiratory organoids, related to Figure 5.

WT ESC (KhES3)-derived respiratory organoids were treated with anti-NCL antibodies (10 µg/mL), anti-ICAM-1 antibodies (10 µg/mL), or isotype control (10 µg/mL). After 1 h of incubation, respiratory organoids were infected with RSV-A at 0.1 TCID/cell (8 × 10^4 TCID/well) and cultured for 96 h. At 96 hpi, the cell culture supernatants of RSV-A-infected WT or antibody-treated respiratory organoids were collected and $TCID_{50}$ assay was performed. Data are shown as mean ± SD (*n*=3, three infection batches); One-way ANOVA with Tukey's post hoc test (**$p < 0.01$).

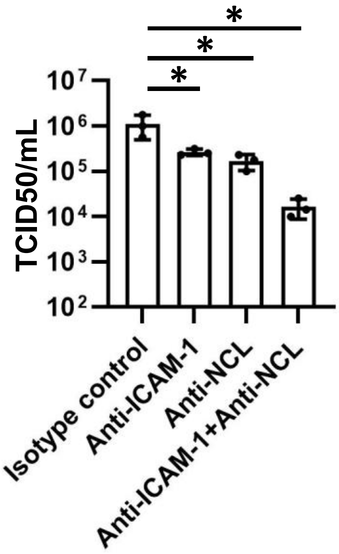

Figure S9. NCL and ICAM-1 play important roles even in high-titer RSV-A infection, related to Figure 5.

WT iPSC-derived respiratory organoids were treated with anti-NCL antibodies (10 μg/mL), anti-ICAM-1 antibodies (10 μg/mL), or isotype control (10 μg/mL). After 1 h of incubation, respiratory organoids were infected with RSV-A at 1.0 TCID/cell (8×10^5 TCID/well) and cultured for 96 h. At 96 hpi, the cell culture supernatants of RSV-A-infected WT or antibody-treated respiratory organoids were collected and $TCID_{50}$ assay was performed. Data are shown as mean ± SD (*n*=3, three infection batches); One-way ANOVA with Tukey's post hoc test (*$p < 0.05$).

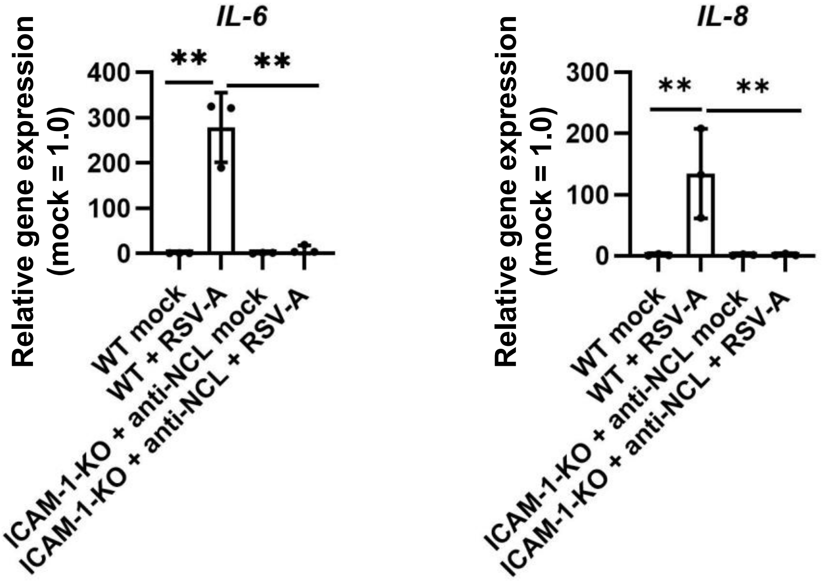

Figure S10. The gene expression levels of inflammatory response-related genes in anti-NCL antibody-treated ICAM-1-KO respiratory organoids, related to Figure 5.

ICAM-1-KO iPSC-derived respiratory organoids were treated with anti-NCL antibodies or an isotype control (10 µg/mL). After 1 h of incubation, respiratory organoids were infected with RSV-A at 0.1 TCID/cell (8 × 10^4 TCID/well) and cultured for 4 days. At 96 hpi, the gene expression levels of *IL-6* and *IL-8* were measured by RT-qPCR in WT, ICAM-1-KO, and anti-NCL antibody-treated ICAM-1-KO respiratory organoids. Data are shown as mean ± SD (n=3, three infection batches); One-way ANOVA with Tukey's post hoc test (**$p < 0.01$).

Table S1. The sequences of gRNA and primers used for genome editing experiments

ICAM-1	gRNA		TGAGGTCACGACCACGGTGCTGG
	Primers for sequencing	Nested PCR (Forward)	CCAAGATCGAGCCACTGTTT
		Nested PCR (Reverse)	TAGGTGACTGTGGGGTTCAA
		Sequencing	GCGTGTTTGGGGGAGATACT
EGFR	gRNA		TGCTGACTATGTCCCGCCACTGG
	Primers for sequencing	Nested PCR (Forward)	CTGGAGTCTACCTGTCTCTGTC
		Nested PCR (Reverse)	GTAGCCTCTGGAGACTCAGAA
		Sequencing	TGCTCAAAGCCCAATTCAGA

Table S2. Primers used for RT-qPCR

Gene name		Sequence
GAPDH	Forward	GGAGCGAGATCCCTCCAAAAT
	Reverse	GGCTGTTGTCATACTTCTCATGG
ICAM1	Forward	ATGCCCAGACATCTGTGTCC
	Reverse	GGGGTCTCTATGCCCAACAA
EGFR	Forward	CCCACTCATGCTCTACAACCC
	Reverse	TCGCACTTCTTACACTTGCGG
NCL	Forward	GGTGGTCGTTTCCCCAACAAA
	Reverse	GCCAGGTGTGGTAACTGCT
IGF1R	Forward	TCGACATCCGCAACGACTATC
	Reverse	CCAGGGCGTAGTTGTAGAAGAG
MX1	Forward	GTTTCCGAAGTGGACATCGCA
	Reverse	CTGCACAGGTTGTTCTCAGC
ISG15	Forward	CGCAGATCACCCAGAAGATCG
	Reverse	TTCGTCGCATTTGTCCACCA
IL6	Forward	ACTCACCTCTTCAGAACGAATTG
	Reverse	CCATCTTTGGAAGGTTCAGGTTG
IL8	Forward	TTTTGCCAAGGAGTGCTAAAGA
	Reverse	AACCCTCTGCACCCAGTTTTC
FOXJ1	Forward	GCCTCCCTACTCGTATGCCA
	Reverse	GCCGACAGGGTGATCTTGG
TUBA1A	Forward	TCGATATTGAGCGTCCAACCT
	Reverse	CAAAGGCACGTTTGGCATACA
MCIDAS	Forward	ATTCCCACCAAACGGAAGCAG
	Reverse	CCAGGGTAGGCGACATCATAG
KLF5	Forward	CCTGGTCCAGACAAGATGTGA
	Reverse	GAACTGGTCTACGACTGAGGC
CYP2F1	Forward	AGCACAGCCATCTTACTCCTG
	Reverse	GGCAGCTTTCCCTTATCTCTTGA
TP63	Forward	GGACCAGCAGATTCAGAACGG
	Reverse	AGGACACGTCGAAACTGTGC
NGFR	Forward	CCTACGGCTACTACCAGGATG
	Reverse	CACACGGTGTTCTGCTTGT
MUC20	Forward	ATGACAACGGACGACACAGAA
	Reverse	TCAGCGTTTGAGTTTCCAGAG
MUC5B	Forward	GCCTACGAGGACTTCAACGTC
	Reverse	CCTTGATGACAACACGGGTGA
Oct3/4	Forward	CTGGGTTGATCCTCGGACCT
	Reverse	CCATCGGAGTTGCTCTCCA
SOX2	Forward	GCCGAGTGGAAACTTTTGTCG
	Reverse	GGCAGCGTGTACTTATCCTTCT
KLF4	Forward	CCCACATGAAGCGACTTCCC
	Reverse	CAGGTCCAGGAGATCGTTGAA
c-MYC	Forward	GGCTCCTGGCAAAAGGTCA
	Reverse	CTGCGTAGTTGTGCTGATGT
NANOG	Forward	TTTGTGGGCCTGAAGAAAACT
	Reverse	AGGGCTGTCCTGAATAAGCAG
RSV-A N	Forward	CATCCAGCAAATACACCATCCA

	Reverse	TTCTGCACATCATAATTAGGAGTATCAA

Table S3. Antibodies used for immunofluorescence staining, capillary-based immunoassay, or flow cytometry

Antigen	Catalog	Host	Company
Goat anti-Mouse IgG (H+L) Highly Cross-Adsorbed Secondary Antibody, Alexa Fluor™ 594	A11032	Goat	Thermo Fisher Scientific
Respiratory Syncytial Virus	AB43812	Mouse	Abcam
OCT3/4	sc-5279	Mouse	Santa Cruz Biotechnology
β-actin	A5441	Mouse	Sigma-Aldrich
ICAM-1	10831-1-AP	Rabbit	Proteintech Group
EGFR	66455-1-Ig	Mouse	Proteintech Group
TRA-1-81	MAB4381	Mouse	Sigma-Aldrich
